# Duration of dual antiplatelet therapy and stability of coronary heart disease: a 60 000-patient meta-analysis of randomised controlled trials

**DOI:** 10.1136/openhrt-2021-001707

**Published:** 2021-08-02

**Authors:** Anda Bularga, Mohammed N Meah, Dimitrios Doudesis, Anoop S V Shah, Nicholas L Mills, David E Newby, Kuan Ken Lee

**Affiliations:** 1 BHF Centre for Cardiovascular Science, University of Edinburgh Division of Clinical and Surgical Sciences, Edinburgh, UK; 2 Department of Non-Communicable Diseases, London School of Hygiene and Tropical Medicine, London, UK; 3 Department of Cardiology, Imperial College Healthcare NHS Trust, London, UK; 4 Usher Institute, University of Edinburgh Division of Health Sciences, Edinburgh, UK

**Keywords:** acute coronary syndrome, angina pectoris, pharmacology, clinical

## Abstract

**Background:**

Dual antiplatelet therapy (DAPT) has important implications for clinical outcomes in coronary disease. However, the optimal DAPT duration remains uncertain.

**Methods and results:**

We searched four major databases for randomised controlled trials comparing long-term (≥12 months) with short-term (≤6 months) or shorter (≤3 months) DAPT in patients with coronary syndromes. The primary outcome was all-cause mortality. Secondary outcomes were any bleeding and major bleeding (safety), cardiac death, myocardial infarction, stent thrombosis, revascularisation and stroke (efficacy). Nineteen randomised controlled trials (n=60 111) satisfied inclusion criteria, 8 assessed ≤3 months DAPT. Compared with long-term (≥12 months), short-term DAPT (≤6 months) was associated with a trend towards reduced all-cause mortality (RR: 0.90, 95% CI: 0.80 to 1.01) and significant bleeding reduction (RR: 0.68, 95% CI: 0.55 to 0.83 and RR: 0.66, 95% CI: 0.56 to 0.77 for major and any bleeding, respectively). There were no significant differences in efficacy outcomes. These associations persisted in sensitivity analysis comparing shorter duration DAPT (≤3 months) to long-term DAPT (≥12 months) for all-cause mortality (RR: 0.91, 95% CI: 0.79 to 1.05). In subgroup analysis, short-term DAPT was associated with lower risk of bleeding in patients with acute or chronic coronary syndromes (RR: 0.66, 95% CI: 0.54 to 0.81 and RR: 0.53, 95% CI: 0.33 to 0.65, respectively), but higher risk of stent thrombosis in acute coronary syndrome (RR: 1.49, 95% CI: 1.02 to 2.17 vs RR: 1.25, 95% CI 0.44 to 3.58).

**Conclusion:**

Our meta-analysis suggests that short (≤6 months) and shorter (≤3 months) durations DAPT are associated with lower risk of bleeding, equivalent efficacy and a trend towards lower all-cause mortality irrespective of coronary artery disease stability.

**Key messages**

**What is already known about this subject?**

- Dual antiplatelet therapy (DAPT) is a central component of the modern management of acute and chronic coronary syndromes following percutaneous coronary intervention. Despite substantial evidence supporting its use, there remains major uncertainty regarding the optimal duration of therapy.

**What does this study add?**

- Short-term (≤6 months) and shorter durations (≤3 months) of DAPT are associated with significantly lower risk of bleeding, equivalent efficacy and a trend towards lower all-cause mortality compared with long-term DAPT (≥12 months) irrespective of coronary artery stability.
- This meta-analysis highlights the paucity of randomised controlled trial evidence to guide DAPT in acute coronary syndrome patients who are managed without percutaneous coronary intervention such as those receiving medical therapy only or those undergoing coronary artery bypass grafting.

**How might this impact on clinical practice?**

- Shorter durations of DAPT in patients with acute or chronic coronary syndrome undergoing percutaneous coronary intervention may be the best balance between efficacy and safety outcomes as shown by all-cause mortality which tended to favour 3 months of therapy.

## Introduction

Dual antiplatelet therapy (DAPT) is a central component of the modern management of acute coronary syndromes (ACS). The aim of DAPT is to reduce the risk of recurrent atherothrombotic events by suppressing thrombus formation related to disrupted atherosclerotic plaque.^1 2^ Despite substantial evidence supporting its use, there remains major uncertainty regarding the optimal duration of therapy. While clinical guidelines on the management of ACS recommend a default duration of 12 months of DAPT with aspirin and a P2Y_12_ receptor antagonist, they also advise consideration of short-term DAPT (≤6 months) for patients at a high risk of bleeding.^3 4^

Previous systematic reviews and meta-analyses concluded that shorter durations of DAPT may be superior to standard care in most patients, with apparent small reductions in all-cause mortality.^5 6^ This suggests that the risk of major bleeding outweighs any benefit gained from the reduction in future atherothrombotic events. These meta-analyses reviewed trials which, for the most part, evaluated DAPT following percutaneous coronary intervention with drug-eluting stents in patients with chronic coronary syndromes. Recently, there have been several large-scale randomised controlled trials evaluating shorter durations of DAPT (≤3 months) in the setting of ACS.^7–9^

Here, we perform an updated systematic review and meta-analysis comparing outcomes in long-term DAPT (≥12 months) with short-term (≤6 months) and shorter (≤3 months) durations of DAPT incorporating the latest randomised controlled trial evidence.

## Methods

### Data sources and search strategy

This systematic review and meta-analysis followed the Cochrane Collaboration guidelines and the Preferred Reporting Items for Systematic Reviews and Meta-Analyses (online supplemental research checklist) and was performed according to a prespecified analysis plan (online supplemental appendix).^10 11^ Two independent investigators (MM and AB) performed the literature search using four major databases: Central, Embase, Medline and Web of Science from 1950 to February 2020. In addition, online resources including ClinicalTrials.gov and proceedings from major cardiovascular conferences were also screened. The search strategy was individually tailored to each database (online supplemental S1 table). Relevant search items such as: ‘coronary syndrome’, ‘antiplatelet therapy’, ‘platelet aggregation inhibitor’, ‘drug eluting stent’, ‘coronary intervention’ were included in the Medical Subject Heading search.

10.1136/openhrt-2021-001707.supp1 Supplementary data

### Study selection

Randomised controlled trials comparing different durations of DAPT, irrespective of presentation (acute or chronic coronary syndromes), or the management strategy (percutaneous coronary intervention, coronary artery bypass graft surgery or medical therapy alone) that assessed at least one of the prespecified outcomes of interest were included in this systematic review and meta-analysis. The DAPT durations of interest were ≤6 months (short-term) versus ≥12 months (long-term). Studies which compared mid-term DAPT (>6 but<12 months) to long-term (≥12 months) or standard term (12 months) to longer-term (>18 months) DAPT were excluded.^12–15^ Cross-sectional studies, observational studies, case reports or case series were also excluded.

### Quality assessment and data extraction

Two investigators (MM and AB) independently screened article titles and abstracts to exclude any trials which did not match the research question of interest. Subsequently, the two reviewers independently screened the eligible full-text articles to identify randomised controlled trials which met the prespecified inclusion criteria. The reference lists of the relevant studies were manually checked to identify potentially missed studies. Data extraction was conducted independently by two authors (MM and AB) and any conflicts related to data extraction were resolved through discussion and review of data or consensus from a third author (KKL).

Data extraction included study characteristics (trial registration number, trial name, trial period, study centre(s), year of publication, first author, randomisation arms (intervention vs control), study population according to randomisation arm, treatment strategy according to randomisation arm, randomisation time, follow-up duration, outcome measures including primary, secondary outcomes and relevant definitions (table 1). Baseline characteristics for study population (age, sex, ACS at presentation, patients with background history of diabetes mellitus, ischaemic heart disease, peripheral vascular disease, renal impairment and cardiovascular risk factors) (online supplemental S2 table) were collected where available, and relevant risk estimates for the primary trial outcome and meta-analysis outcomes of interest (online supplemental S3 table).

**Table 1:** Study characteristics according to randomisation arm

Study	DAPT duration (months)	Total population	ACS population	CCS population	DAPT regimen	Randomisation	Follow-up	Primary outcome
CREDO* (Steinhubl *et al*^39^ 2002)	1	1063	703 (66%)	360 (34%)	Aspirin 81–325 mg plus clopidogrel 75 mg	Prior to index PCI	12 months	Composite of death, myocardial infarction (MI) and stroke in the intention-to-treat population.
	12	1053	704 (67%)	349 (33%)	Aspirin 81–325 mg plus placebo			
DAPT-STEMI (Kedhi *et al*^40^ 2018)	6	433	433 (100%)	0 (0%)	Aspirin 75–100 mg	Six months following index PCI	24 months	Composite of all-cause mortality, any MI, any revascularisation, stroke or thrombolysis.
	12	437	437 (100%)	0 (0%)	Aspirin 75–100 mg plus prasugrel 10 mg or 5 mg/ticagrelor 90 mg/clopidogrel 75 mg			
EXCELLENT (Gwon *et al*^41^ 2012)	6	722	369 (51%)	353 (49%)	Aspirin 100–200 mg	At index PCI	12 months	Composite of cardiac death, MI or target vessel revascularisation.
	12	721	375 (52%)	346 (48%)	Aspirin 100–200 mg plus clopidogrel 75 mg			
GLOBAL LEADERS* (Vranckx *et al*^7^ 2018)	1	7980	3750 (47%)	4230 (53%)	Ticagrelor 90 mg	At index PCI	24 months	Composite of all-cause death or new Q-wave MI.
	12	7988	3737 (47%)	4251 (53%)	Ticagrelor 90 mg or clopidogrel 75 mg and aspirin 75–100 mg			
I-LOVE-IT-2 (Han *et al*^42^ 2016)	6	909	752 (83%)	157 (17%)	Aspirin 100 mg	At index PCI	18 months	Target lesion failure.
	12	920	744 (81%)	176 (19%)	Aspirin 100 mg and clopidogrel 75 mg			
ISAR-SAFE (Schulz-Schupke *et al*^43^ 2015)	6	1997	794 (40%)	1203 (60%)	Aspirin 81–162 mg	Six months after index PCI	9 months	Composite of death, MI, stent thrombosis (definite or probable), stroke or thrombolysis in myocardial infarction (TIMI) major bleeding.
	12	2003	807 (40%)	1196 (60%)	Aspirin 81–162 mg combined with clopidogrel 75 mg or ticlopidine 200 mg			
ITALIC (Didier *et al*^44^ 2017)	6	926	400 (43%)	526 (57%)	Aspirin 75 mg	Six months following index PCI	24 months	Composite of all-cause mortality, MI, target vessel revascularisation, stroke, major bleeding, stent thrombosis.
	24	924	406 (44%)	518 (56%)	Aspirin 75 mg and clopidogrel 75 mg or prasugrel 60 mg or ticagrelor 90 mg			
IVUS-XPL (Hong *et al*^45^ 2016)	6	699	343 (49%)	356 (51%)	Aspirin 100 mg	At index PCI	12 months	Composite of cardiac death, MI, stroke or TIMI major bleeding.
	12	701	343 (49%)	358 (51%)	Aspirin 100 mg plus clopidogrel 75 mg			
NIPPON (Nakamura *et al*^46^ 2017)	6	1654	527 (32%)	1127 (68%)	Aspirin 81–162 mg	At index PCI	18 months	Net adverse clinical and cerebrovascular events defined as all cause death, Q-wave or non-Q-wave MI, cerebrovascular events, and major bleeding events.
	18	1653	552 (33%)	1101 (67%)	Aspirin 81–162 mg combined with clopidogrel 75 mg or ticlopidine 200 mg			
OPTIMA-C (Lee *et al*^47^ 2018)	6	684	348 (51%)	336 (49%)	Aspirin 100 mg	At index PCI	12 months	Composite of major adverse cardiovascular events (MACCE; cardiac death, target vessel-related MI, ischaemia driven target lesion revascularisation.
	12	683	344 (50%)	339 (50%)	Aspirin 100 mg plus clopidogrel 75 mg			
OPTIMIZE* (Feres *et al*^48^ 2013)	3	1563	494 (32%)	1069 (68%)	Aspirin 100–200 mg	At index PCI	12 months	Composite of death from all causes, MI, stroke or major bleeding.
	12	1556	502 (32%)	1054 (68%)	Aspirin 100–200 mg plus clopidogrel 75 mg			
PRODIGY (Valgimigli *et al*^49^ 2012)	6	983	733 (75%)	250 (25%)	Aspirin 80–160 mg	One month after index PCI	24 months	Composite of death of any cause, nonfatal MI or cerebrovascular accident; cardiovascular death, the incidence of stent thrombosis and bleeding outcomes.
	24	987	732 (74%)	255 (26%)	Aspirin 80–160 mg plus clopidogrel 75 mg			
REDUCE* (De Luca *et al*^50^ 2019)	3	733	733 (100%)	0 (0%)	Aspirin	At index PCI	24 months	Composite of all-cause death, MI, stent thrombosis, stroke, target vessel revascularisation, bleeding.
	12	727	727 (100%)	0 (0%)	Aspirin and P2Y_12_ inhibitor (prasugrel, ticagrelor or clopidogrel)			
RESET* (Kim et al^51^ 2012)	3	1059	588 (56%)	471 (44%)	Aspirin 100 mg	At index PCI	12 months	Composite of death from cardiovascular cause, MI, stent thrombosis, ischaemia driven target-vessel revascularisation or bleeding.
	12	1058	568 (54%)	490 (46%)	Aspirin 100 mg and clopidogrel 75 mg			
SECURITY* (Colombo *et al*^52^ 2014)	6	682	213 (31%)	469 (69%)	Aspirin	At index PCI	24 months	Composite of cardiac death, MI, stroke, definite or probable stent thrombosis, BARC 3 or 5 bleeding, target vessel revascularisation, all-cause mortality.
	12	717	229 (32%)	488 (68%)	Aspirin plus clopidogrel 75 mg			
SMART-DATE (Hahn *et al*^9^ 2018)	6	1357	1357 (100%)	0 (0%)	Aspirin 100 mg	At index PCI	18 months	Composite of all-cause mortality, MI or stroke.
	12	1355	1355 (100%)	0 (0%)	Aspirin 100 mg and clopidogrel 75 mg, prasugrel 10 mg or ticagrelor 90 mg			
STOPDAPT-2* (Watanabe *et al*^53^ 2019)	1	1500	565 (38%)	935 (62%)	Clopidogrel 75 mg	At index PCI	12 months	Composite of cardiovascular and bleeding events (cardiovascular death, MI, definite stent thrombosis, ischaemic or haemorrhagic strokeor TIMI major or minor bleeding.
	12	1509	583 (39%)	926 (61%)	Aspirin 81–200 mg and clopidogrel 75 mg or prasugrel 3.75 mg			
TICO* (Kim *et al*^25^ 2020)	3	1527	1527 (100%)	0 (0%)	Ticagrelor 90 mg	At index PCI	12 months	Net adverse clinical events (TIMI major bleeding and MACCE).
	12	1529	1529 (100%)	0 (0%)	Ticagrelor 90 mg and aspirin			
TWILIGHT* (Mehran *et al*^8^ 2019)	3	3555	2273 (56%)	1282 (44%)	Ticagrelor 90 mg plus placebo	At index PCI	12 months	The first occurrence of BARC type 2, 3 or 5 bleeding between randomisation and 1 year in a time-to-event analysis.
	12	3564	2341 (66%)	1223 (34%)	Ticagrelor 90 mg and aspirin			

*Trials included in the sensitivity analysis (compared ≤3 months of DAPT with 12 months of DAPT).

ACS, acute coronary syndrome; BARC, Bleeding Academic Research Consortium; CCS, chronic coronary syndrome; DAPT, dual antiplatelet therapy; MACCE, major adverse cardiac and cerebrovascular events; MI, myocardial infarction; PCI, percutaneous coronary intervention; TIMI, thrombolysis in myocardial infarction.

The study quality was assessed using the Cochrane Collaboration tool for assessment of risk of bias, which includes random sequence generation; allocation concealment; blinding of participants, personnel and outcome assessors; incomplete outcome data; selective reporting and other sources of bias.^16^ Disagreements were resolved by consensus.

### Definition of outcomes

The primary outcome was all-cause mortality and secondary efficacy outcomes were cardiac death, myocardial infarction, stent thrombosis, coronary revascularisation and stroke. Secondary safety endpoints were any bleeding and major bleeding. Stent thrombosis included definite or probable thrombosis according to individual trial definitions and criteria from the Academic Research Consortium.^17^ Trial definitions for major and any bleeding were applied, and these included the Thrombolysis in Myocardial Infarction or Bleeding Academic Research Consortium criteria (online supplemental S4 table).^18 19^ Randomised controlled trials which did not report event rates or risk estimates for the prespecified endpoints were not included in the overall meta-analysis estimates.

### Statistical analysis

In this pairwise meta-analysis, risk estimates and event rates for each outcome of interest were extracted from the randomised controlled trials. Risk ratios and 95% CI were used as summary statistics to evaluate the effect of DAPT duration on the outcomes of interest. Pooled meta-analysis risk estimates were computed using a random-effects model. Risk ratios greater than one represented benefit associated with the longer DAPT duration arm (control), and less than one was associated with benefit favouring the shorter duration arm (intervention).

Between study heterogeneity was assessed using the statistical inconsistency test (I^2^=100% × (Q−df)/Q, where Q= χ^2^ (Cochran’s heterogeneity statistic) and df=its degrees of freedom), where I*^2^* ≤25% signifies low heterogeneity, I^2^ ≤50% is moderate heterogeneity and I*^2^* >50% is considered high heterogeneity.^20^ Small study effects and potential publication bias were examined by constructing funnel plots for the clinical outcomes in which the SE of the log of the risk ratio was plotted against the risk ratio (central estimate).^21^

Sensitivity analyses restricted to trials evaluating shorter durations of DAPT (≤3 months) were conducted to explore the primary outcome of all-cause mortality and the secondary efficacy and safety outcomes. A further sensitivity analysis was conducted to explore the effect of the type of P2Y_12_ inhibitor on study outcomes, restricting analysis to trials that used clopidogrel only or to studies that used any type of P2Y_12_ inhibitor (clopidogrel or prasugrel or ticagrelor). Subgroup analysis evaluating the effect of clinical presentation was also performed from data in trials that reported risk ratios stratified by clinical presentation. ACS was defined as patients suspected of acute myocardial infarction/ischaemia, and chronic coronary syndromes was defined as patients with stable symptoms of coronary artery disease.

Analysis was performed using R V.3.5.0 (R Foundation for Statistical Computing, Vienna, Austria) using the *meta, metafor* and *metaviz* packages.

### Role of the funding source

The funder of the study had no role in study design, data collection, data analysis, data interpretation or writing of the report. The corresponding author had full access to all of the data and the final responsibility to submit for publication.

## Results

Our systematic search identified 44 424 articles and 28 863 underwent title and abstract screening after duplicates were removed (online supplemental S1 figure). Of these, 46 potentially eligible articles underwent full-text review, and a further 27 articles were excluded based on pre-specified criteria. A total of 19 randomised controlled trials from 2001 to 2018 with an overall population of 60 111 patients (ranging from 870 to 15 968 in individual studies) were included. Of the total population, 33 952 (56%) were ACS and 26 159 (44%) were chronic coronary syndromes. Four randomised controlled trials evaluated duration of DAPT in ACS exclusively (n=8098), while 15 trials included both acute and chronic presentations. No randomised controlled trial investigating duration of DAPT in patients with ACS managed medically or undergoing coronary artery bypass graft surgery were identified.

The duration of DAPT across trials ranged from 1 month to 24 months. Duration of follow-up also varied between trials ranging from 9 months to 24 months. Of the 19 included randomised controlled trials, eight trials compared shorter-term DAPT (≤3 months) with long-term DAPT (>12 months) with an overall population of 38 036 patients and two of these studies included ACS presentations only (Table 1 and online supplemental S3 table).

### Risk of bias and publication bias

The risk of bias assessment was performed for each randomised controlled trial (online supplemental S4 table). All studies were assessed as having low risk of bias for random sequence allocation (19/19, 100%) with majority of studies being low risk for allocation concealment (15/19, 79%), blinding of outcome assessment (15/19, 79%), incomplete outcome data (18/19, 95%), selective reporting (17/19, 90%) and other bias (18/19, 95%). The majority of studies were identified to be at risk of bias due to inadequate blinding of participants and personnel (16/19, 84%). Allocation concealment was unclear in 3/19 (16%) studies. Evaluation of the funnel plots suggests a degree of publication bias when considering the safety outcomes of any bleeding and major bleeding (online supplemental S2 figure).

### Short-term (≤6 months) versus long-term (≥12 months) dual antiplatelet therapy

**Figure 1:**
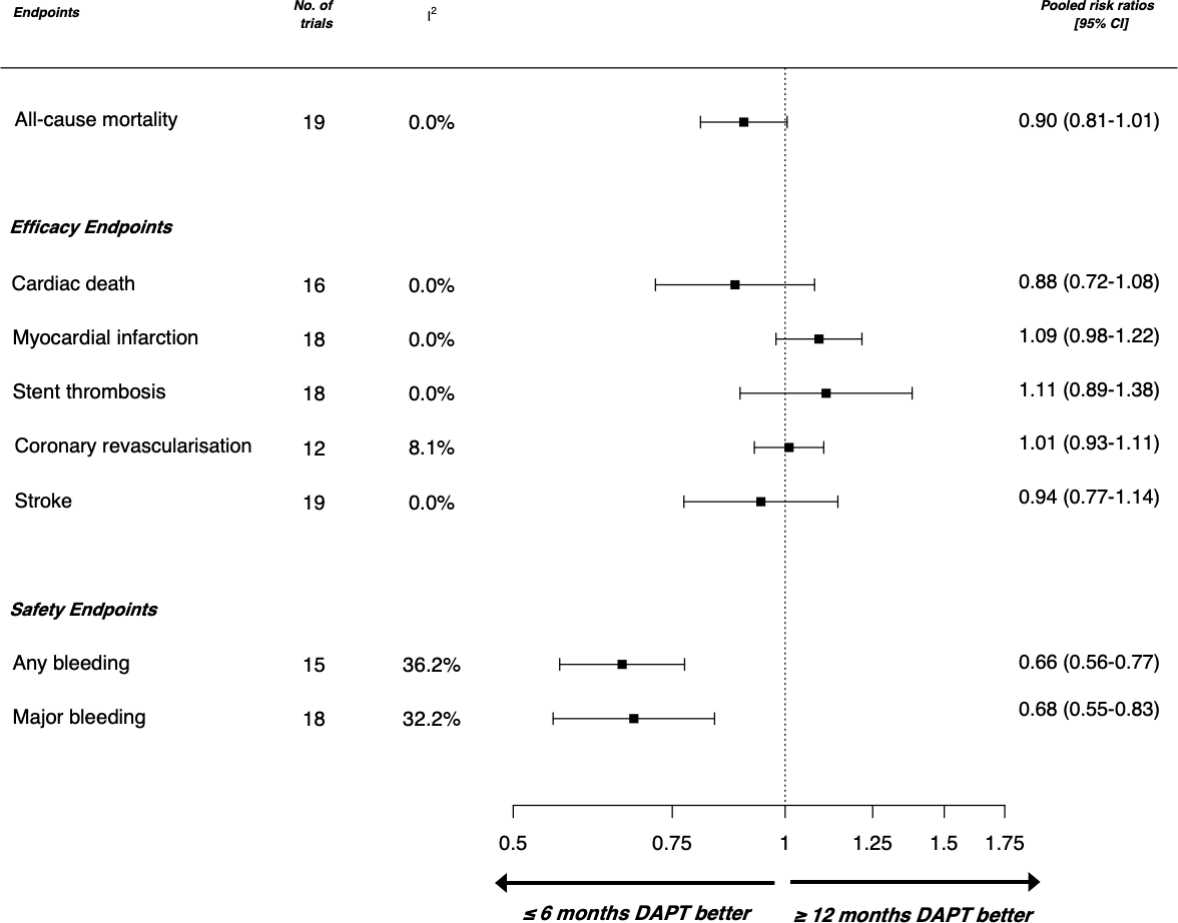
Forest plot showing overall pooled risk estimates according to outcomes of interest from all randomised controlled trials comparing short duration of dual-antiplatelet therapy (≤6 months) and long duration (≥12 months) included in this meta-analysis (n=60 111). DAPT, dual antiplatelet therapy.

All 19 randomised trials reported the primary outcome of all-cause mortality. Short-term DAPT was associated with an apparent decrease in all-cause mortality (RR: 0.90, 95% CI: 0.81 to 1.01) (figure 1). There was no significant heterogeneity between studies when considering all-cause mortality (I^2^=0%). Individual trial data are presented in online supplemental S3 figure. A similar trend towards reduced all-cause mortality was observed with short-term DAPT in trials (n=8) which used different P2Y_12_ receptor antagonists including clopidogrel, ticagrelor or prasugrel (RR: 0.87, 95% CI: 0.76 to 1.00). While in an analysis restricted to studies (n=11) that used clopidogrel only as the P2Y_12_ receptor antagonist, the pooled risk estimates for all-cause mortality were equivalent (RR: 0.97, 95% CI: 0.80 to 1.18) when considering DAPT duration online supplemental S4 figure.

All studies reported the efficacy end point of stroke, 18 studies evaluated the secondary endpoints of myocardial infarction and stent thrombosis, 16 and 12 studies reported cardiac mortality and coronary revascularisation, respectively. A trend towards increased risk of myocardial infarction (RR: 1.09, 95% CI: 0.98 to 1.22) and equivalent risk of stent thrombosis (RR: 1.11, 95% CI: 0.89 to 1.38) and coronary revascularisation (RR: 1.01, 95% CI: 0.93 to 1.11) was observed with short-term DAPT when compared with long-term DAPT (≥12 months). Short-term DAPT was associated with similar risk of cardiac mortality (RR: 0.88, 95% CI: 0.72 to 1.08) and stroke (RR: 0.94, CI: 95% 0.77 to 1.14). There was no significant heterogeneity between studies when considering these efficacy outcomes (I^2^ <25%). Individual trial data are presented in online supplemental S3 figure.

Of the 19 studies, 18 reported the safety endpoint of major bleeding and 15 reported ‘any bleeding events’. Study-specific definitions are summarised in online supplemental S4 table. Short-term DAPT was associated with a reduction in bleeding when compared with long-term DAPT, with RR of 0.68 (95% CI: 0.55 to 0.83) for major bleeding and RR: 0.66 (95% CI: 0.56 to 0.77) for any bleeding. Modest heterogeneity (I^2^*=*32.2%) was observed across the studies when assessing these safety outcomes. Individual trial data are presented in online supplemental S3 figure.

### Shorter duration (≤3 months) versus long-term (≥12 months) dual antiplatelet therapy

**Figure 2:**
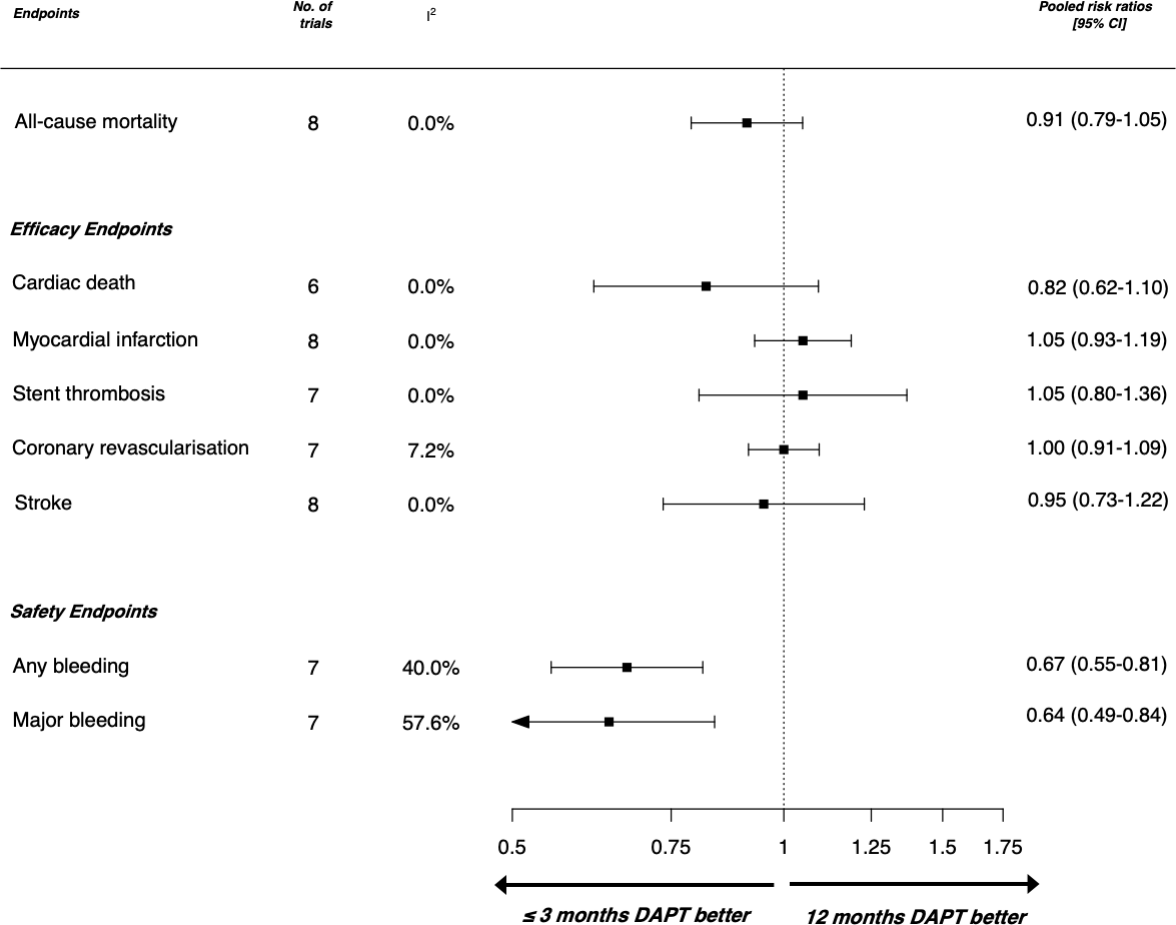
Forest plot showing pooled risk estimates according to outcomes of interest restricted to randomised controlled trials comparing even shorter duration of dual-antiplatelet therapy (≤3 months) and standard duration (12 months) included in this meta-analysis (n=38 036) DAPT, dual antiplatelet therapy.

Meta-estimates were consistent in sensitivity analysis restricted to the eight trials comparing shorter durations of DAPT with long-term DAPT. The trend towards a reduction in all-cause mortality was maintained with shorter duration DAPT (RR: 0.91, 95% CI: 0.79 to 1.05) with no significant heterogeneity across the studies (I^2^*=*0%) (figure 2).

The pooled risk meta-estimates were equivalent for myocardial infarction (RR: 1.05, 95% CI: 0.93 to 1.19), stent thrombosis (RR: 1.05, 95% CI: 0.8 to 1.36), repeat revascularisation (RR: 1.0, 95% CI: 0.91 to 1.09) and stroke (RR: 0.95, 95% CI: 0.73 to 1.22). In shorter duration DAPT, estimates appeared to suggest a lower risk for cardiac death (RR: 0.82, 95% CI: 0.62 to 1.1). There was no significant heterogeneity between studies when considering these efficacy outcomes (I^2^ <10%).

Of the eight trials, seven reported results on major bleeding and any bleeding events. The observed reduction in major bleeding was maintained with shorter duration DAPT when compared with long-term DAPT (RR: 0.64, 95% CI: 0.49 to 0.84). There was however high heterogeneity observed across these studies (I^2^=57.6%).

### Duration of DAPT in acute or chronic coronary syndromes

**Figure 3:**
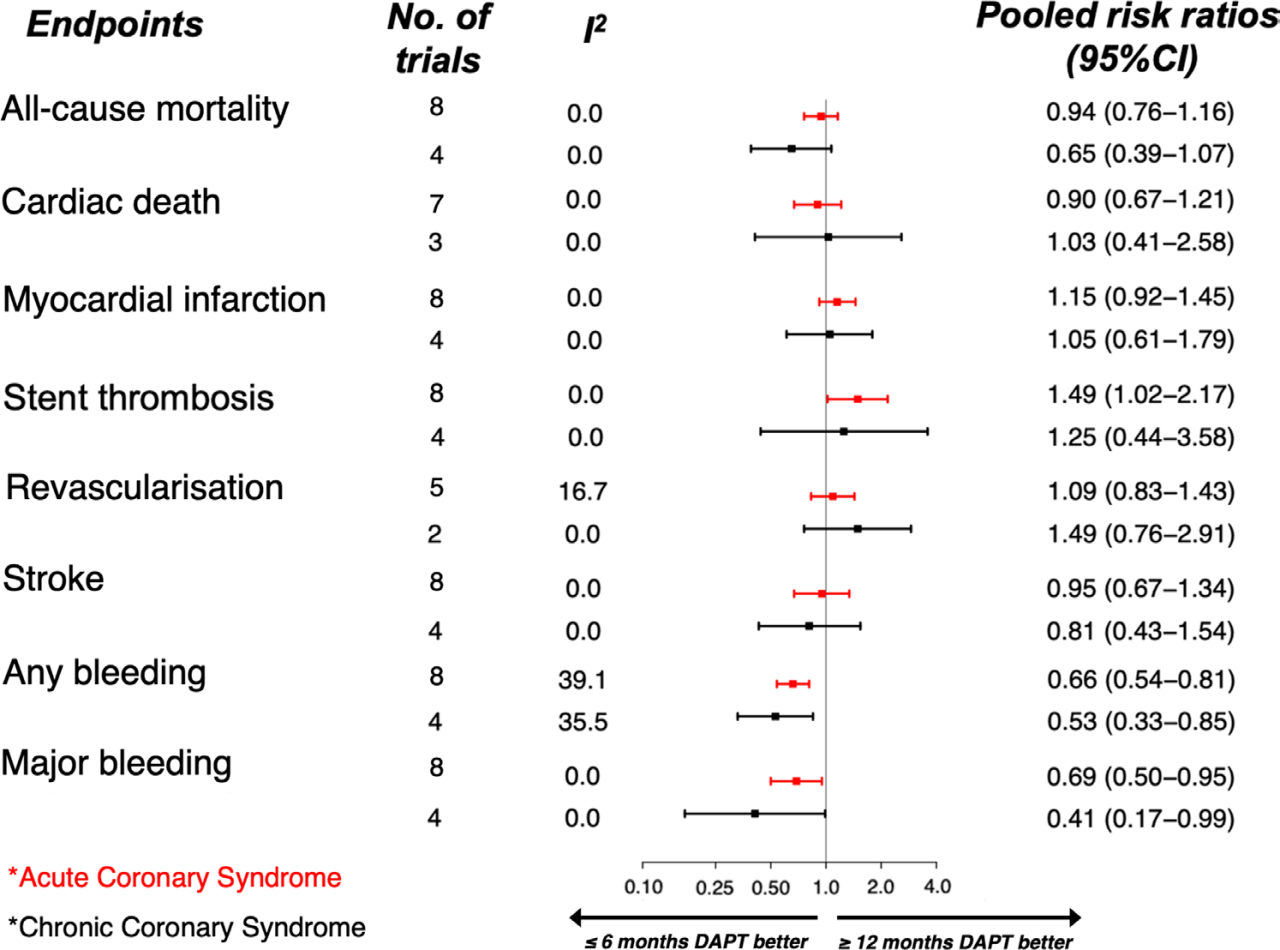
Forest plot showing pooled risk estimates according to outcomes of interest in subgroups of patients with acute coronary syndrome (red) (n=13 466) and chronic coronary syndrome (black) (n=4281) comparing short duration of dual-antiplatelet therapy (≤6 months) and long duration (12 months); (DAPT, dual antiplatelet therapy).

Subgroup meta-analyses revealed a trend towards reduced risk of all-cause mortality with shorter duration DAPT in patients in whom the index presentation was ACS (RR: 0.94, 95% CI: 0.76 to 1.16) and towards further reduced risk in those with chronic coronary syndrome (RR: 0.65, 95% CI: 0.39 to 1.07). Risk estimates did not differ across the majority of efficacy outcomes with cardiac death, myocardial infarction, repeat revascularisation and stroke demonstrating equivalent risk ratios regardless of presentation (figure 3). There was an apparent increased risk of stent thrombosis in patients on shorter durations of DAPT presenting with ACS (RR: 1.49, 95% CI: 1.02 to 2.17 for ACS and RR: 1.25, 95% CI: 0.44 to 3.58 for chronic coronary syndromes). There was no significant heterogeneity between studies when considering these outcomes (I^2^ <25%). Short duration DAPT was associated with a reduction in bleeding across subgroups, both for major bleeding (ACS RR: 0.69, 95% CI: 0.5 to 0.95, and chronic coronary syndrome RR: 0.41, 95% CI: 0.17 to 0.99) and any bleeding (ACS RR: 0.66, 95% CI: 0.54 to 0.81, and chronic coronary syndromes RR: 0.53, 95% CI: 0.33 to 0.65).

## Discussion

We here report a systematic review and meta-analysis of 19 randomised controlled trials evaluating the efficacy and safety of short-term DAPT compared with long-term DAPT. Our principal finding suggests a trend towards a reduced risk of all-cause mortality in patients who had short-term DAPT. This was true even when duration of therapy was reduced from ≤6 months to ≤3 months of DAPT, with no apparent increase in atherothrombotic events. Moreover, these observations were consistent when comparing patients who presented with acute or chronic coronary syndromes, with the exception of stent thrombosis where an increased risk was noted in those on shorter durations of DAPT for patients presenting acutely. These findings highlight the uncertainty regarding current guideline recommendations for a default strategy of 12 months of DAPT in patients with ACS.^3 4^

In a meta-analysis of 10 trials of patients with chronic coronary syndrome undergoing percutaneous coronary intervention, Palmerini and colleagues suggested that, while 6 months of DAPT resulted in increased rates of myocardial infarction and stent thrombosis, this did not translate into a reduction in cardiovascular death when compared with 12 months of therapy.^22^ However, they observed lower all-cause mortality with the use of short-term DAPT driven by a lower risk of major bleeding and significant reduction in non-cardiovascular death. As a result of this meta-analysis, the European Society of Cardiology and the American Heart Association/American College of Cardiology guidance changed for patients with chronic coronary syndromes who underwent percutaneous coronary intervention to make 6 months of DAPT the standard of care. These observations did not influence recommendations on the duration of DAPT in ACS where these guidelines continue to recommend a 12-month duration of therapy as standard of care.^3 4^

Yin and colleagues recently published a network meta-analysis comparing short-term (<6 months) with standard term (12 months) and longer-term (≥18 months) DAPT.^6^ Their analysis included 17 studies and also reported a reduction all-cause mortality and fewer bleeding events in patients on short-term DAPT, despite including more studies that had enrolled patients with ACS. Their sensitivity analysis comparing patients by acute or chronic presentation, demonstrated short-term DAPT had equivalent safety and efficacy outcomes when compared with longer durations. Khan and colleagues conducted a network meta-analysis of 24 trials on patients requiring DAPT following percutaneous coronary intervention, which additionally compared outcomes in those on mid-term DAPT (6–12 months). They reported equivalent outcomes for all-cause mortality across groups, though a trend towards reduced risk in patients on short-term DAPT was noted. While risk ratios for myocardial infarction were reduced in long-term DAPT, this was again counter-balanced by an increase in bleeding events.^23^ Even in high-risk patients with diabetes mellitus, meta-analyses suggest equivalent rates of all-cause mortality, cardiac death and adverse cardiac events regardless of duration of DAPT.^24^ Our report is consistent with these recent meta-analyses. However, we have here included newer trials such as Kim *et al* and Mehran *et al*, which assessed shorter term DAPT (<3 months vs ≥12 months).^8 25^ In our analysis, shorter duration of DAPT (≤3 months) was associated with a trend towards lower all-cause mortality, remained similarly effective in key efficacy outcomes, but had substantially lower rates of bleeding when compared with long-term DAPT (≥12 months). While majority of trials evaluating duration of DAPT used clopidogrel, more recent trials have evaluated potent P2Y_12_ receptor antagonists.^8 25^ Similar to findings from Navarese *et al*, we observed a reduction in all-cause mortality with shorter durations of therapy in studies including potent P2Y_12_ receptor antagonists compared with those that used clopidogrel only.^26^

Why should we consider 3 months of DAPT to be any different to 6 months of DAPT? Multiple trials in the patients with ACS have demonstrated high initial ischaemic event rates which revert to lower linear rates from 3 months onwards.^1 2 27 28^ Consequently, the largest absolute reductions in cardiovascular events are driven by the use of DAPT in the first 3 months after an ACS. Indeed, in the CURE trial, DAPT caused the majority of the reductions in recurrent myocardial infarction within the first 3 months with only modest benefits thereafter.^29^ In contrast, there was a persistent and continuous bleeding hazard that was not time dependent, suggesting that the prevention of myocardial infarction may become counterbalanced by the hazards of bleeding beyond 3 months.^29^

Withdrawal of P2Y_12_ receptor antagonists from DAPT is associated with a rebound prothrombotic effect and is associated with an increase in rates of stent thrombosis.^30^ We observed this phenomenon, especially in those with ACS randomised to a shorter duration of DAPT. Stent thrombosis does however occur irrespective of the timing of withdrawal as demonstrated in the DAPT trial where rebound stent thrombosis was seen after DAPT cessation at both 12 and 30 months.^13^ This perhaps emphasises the importance of procedural variables, such as optimal stent deployment especially in patients with ACS when deciding on the duration of DAPT. As such, a small but persistent risk of stent-thrombosis will persist when transitioning from dual therapy to monotherapy whenever this occurs.

It should be noted that stent thrombosis occurs infrequently and did not correlate with increased mortality. Advances in stent technologies have reduced rates of stent thrombosis.^31^ Bleeding events on the other hand occur much more often, and the subsequent risk of all-cause mortality has been demonstrated in a wide variety of trials in patients with coronary artery disease regardless of trial intervention. For example, in trials of anticoagulant therapy use in ACS, those therapies with a lower bleeding hazard have a lower all-cause mortality despite having similar efficacy in preventing atherothrombotic events.^32^ Moreover, trials of arterial access sites for percutaneous coronary intervention in ACS also demonstrate a mortality benefit that is attributable to lower rates of bleeding with radial artery access.^33 34^ This supports the notion that bleeding events are an important determinant of all-cause mortality in patients receiving treatment for coronary artery disease and consequently, therapeutic approaches that minimise the risk of bleeding have the potential to reduce mortality in these patients.

Our systemic review and meta-analysis highlight the paucity of randomised controlled trial evidence to guide DAPT in patients with ACS who are managed without percutaneous coronary intervention such as those receiving medical therapy only or those undergoing coronary artery bypass grafting. Patients with ACS who are managed with medical therapy only are often at the extremes of risk with either an event attributable to minor coronary artery disease or multiple comorbidity and a contraindication to invasive coronary angiography. Registry data suggest between 20% and 40% of all admissions for non-ST segment elevation myocardial infarctions are managed medically and recurrent events can be as much as three times more likely to occur in this population.^35 36^ The balance of bleeding and ischaemic risk is clearly challenging in these situations. For patients who are treated with coronary artery bypass graft surgery, DAPT is only offered to those with ACS, and following a brief interruption for surgery, are usually maintained on therapy for 12 months. Bleeding and ischaemic risk in these patients are likely to be affected by the surgical procedure itself and therefore they represent a group that is distinct from other patients with acute or chronic coronary syndromes. While meta-analyses show DAPT prevents graft occlusion, none have robustly assessed the optimum duration of therapy.^37^

It is important to acknowledge that randomised controlled trials rightly have strict entry and exclusion criteria for their study participants. Patients with bleeding risks have been systematically excluded from these randomised controlled trials which report lower rates of bleeding and non-cardiovascular mortality than the general population.^38^ However, in real-world practice, clinicians make individual decisions with their patients on whether to initiate DAPT and this may include those who would otherwise not have been entered into clinical trials because of a history of bleeding. There is, therefore, a real concern that bleeding risk may be under appreciated and bleeding events may be disproportionately greater with the wider use of DAPT in clinical practice. As such, we believe that there is a clear and pressing need to address what the optimum duration of DAPT is in a broad and unselected cohort of patients suffering ACS. Major randomised controlled trials, such as Duration of Dual Antiplatelet Therapy in Acute Coronary Syndrome (DUAL-ACS2), may help answer this question (NCT03252249).

We should acknowledge the limitations of our meta-analysis. First, the data were gathered, and conclusions drawn from study-level data, and the majority of trials included were designed to test for non-inferiority. Individual patient-level data may have added further insights particularly when considering clinical presentations. Time to randomisation varied across the trials, as did duration of follow-up, which may affect the robustness of overall results. Different antiplatelet combinations were used, some with more potent P2Y_12_ receptor antagonists than others, and some discontinuing aspirin rather than P2Y_12_ receptor antagonists at the end of the DAPT treatment period. The data gathered for our analysis of DAPT in ACS are derived mostly from subgroup analyses and may not be reflective of ‘real-world higher risk’ populations. As such, care should be taken when interpreting the results. Additionally, the majority of trials included were deemed to be at risk of bias due to inadequate blinding of participants and personnel. Finally, endpoint definitions varied across the studies leading to increased heterogeneity particularly when considering bleeding outcomes.

In conclusion, our systematic review and meta-analysis suggest that short-term (≤6 months) and shorter durations (≤3 months) of DAPT are associated with lower risk of bleeding, equivalent efficacy and a trend towards lower all-cause mortality. There remains major uncertainty about the optimal duration of DAPT that requires to be resolved in future trials, particularly for patients with ACS, and those managed without percutaneous coronary intervention.

## Data Availability

All data relevant to the study are included in the article or uploaded as supplementary information. Data tables and analysis code can be made available upon reasonable request to the corresponding author.
