# Supplementary material for: Duration of dual antiplatelet therapy and stability of coronary heart disease: a 60 000-patient meta-analysis of randomised controlled trials
Source: Open Heart. 2021 Aug 2;8(2):e001707. doi: 10.1136/openhrt-2021-001707 (PMC8330558; doi:10.1136/openhrt-2021-001707)
Supplement: Supplementary data [file openhrt-2021-001707supp001.pdf]

SUPPLEMENTAL MATERIAL

Duration of dual antiplatelet therapy and stability of coronary heart disease: a 60,000-patient meta-analysis of randomised controlled trials

Anda Bularga M.D.,[1*] Mohammed N. Meah M.D.,[1*] Dimitrios Doudesis MSc,[1]
Anoop SV Shah M.D.,[2,3] Nicholas L Mills M.D.,[1,4] David E Newby M.D.,[1]
Kuan Ken Lee M.D.[1]

[1]*BHF Centre of Cardiovascular Science, University of Edinburgh*

[2] *Department of Non-communicable Diseases, London School of Hygiene and Tropical Medicine, London, UK*

[3]*Department of Cardiology, Imperial College Healthcare NHS Trust, London, UK*

[4]*Usher Institute of Population Health Sciences and Informatics, University of Edinburgh, United Kingdom*

*Joint first authors, equal contributions

S1 Figure. PRISMA diagram

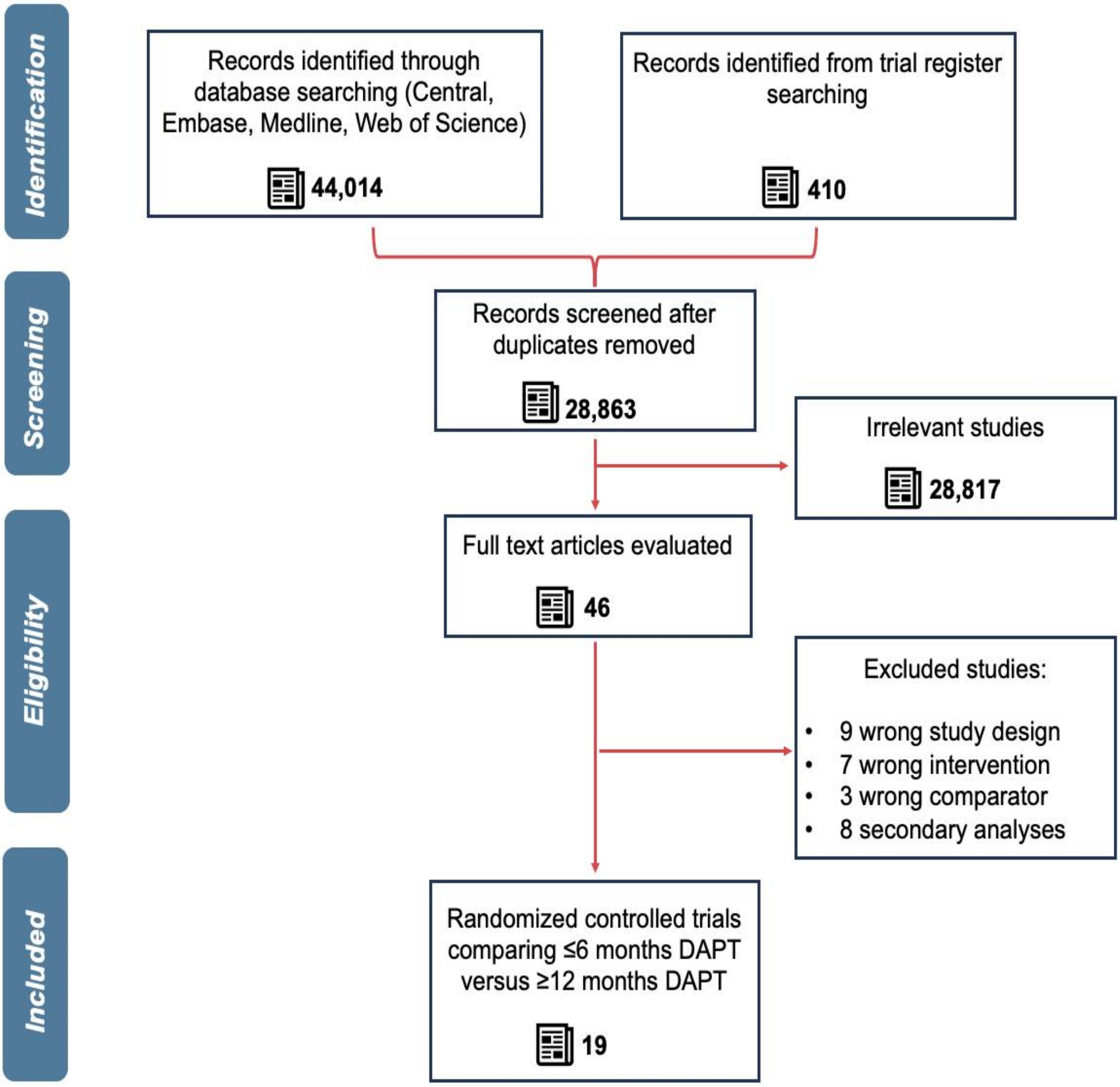

S2 Figure. Funnel plots evaluating publication bias according to outcomes of interest

- ■ RCTs ≤3 months DAPT *vs* 12 months DAPT
- ● RCTs ≤6 months DAPT vs ≥ 12 months DAPT

A. *All-cause mortality*

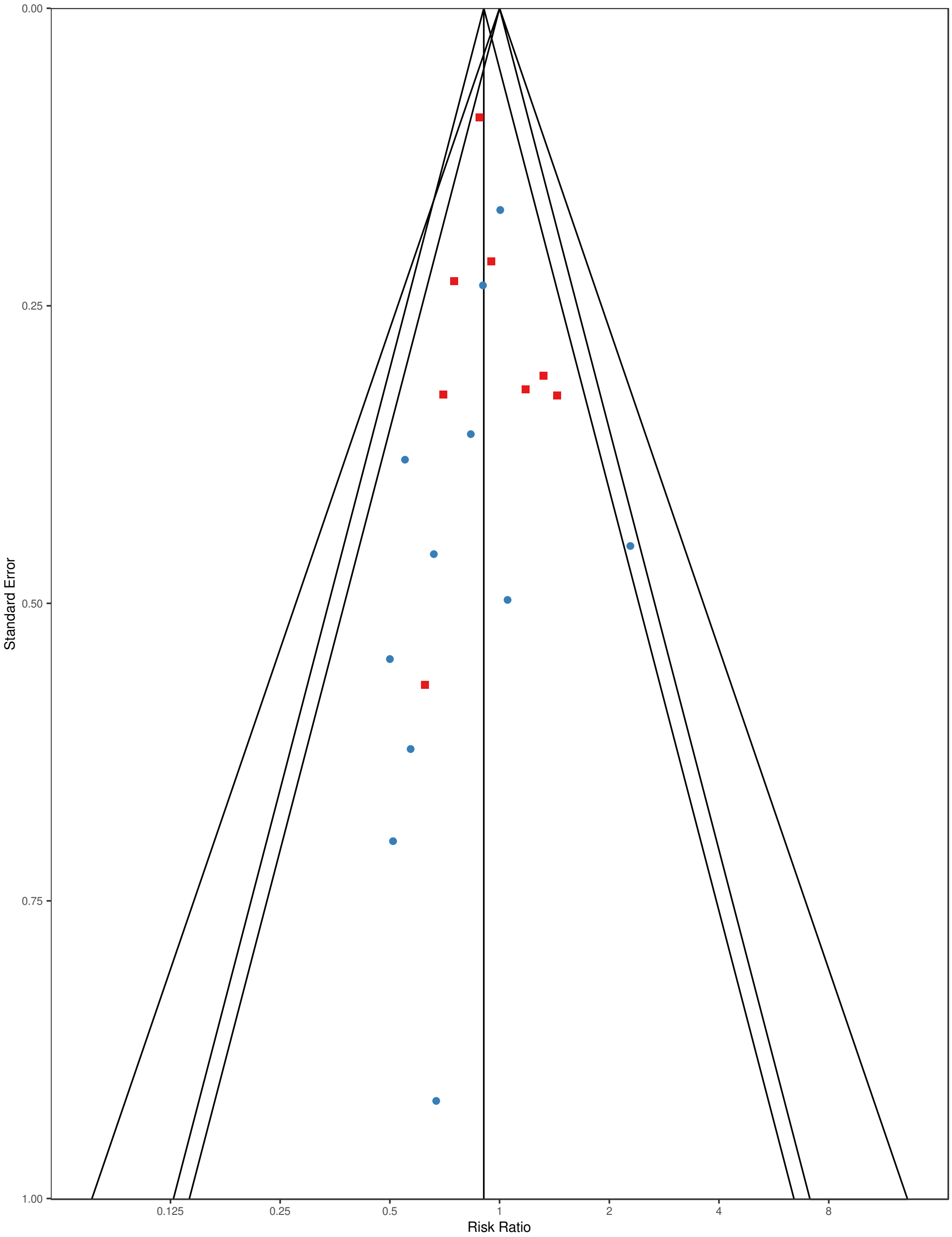

Bularga A, *et al. Open Heart* 2021; 8:e001707. doi: 10.1136/openhrt-2021-001707

B. Efficacy Endpoint:
Cardiac death

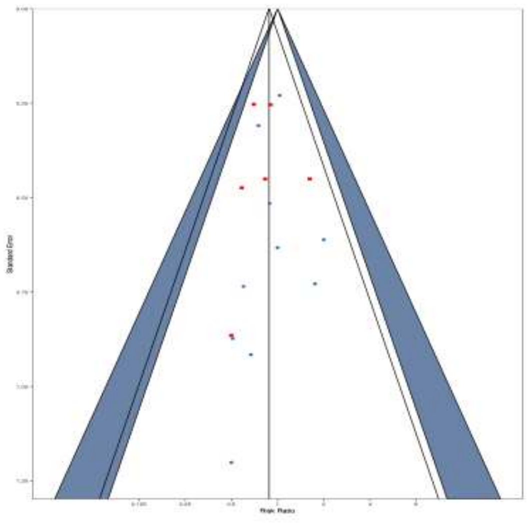

C. Efficacy Endpoint:
Myocardial infarction

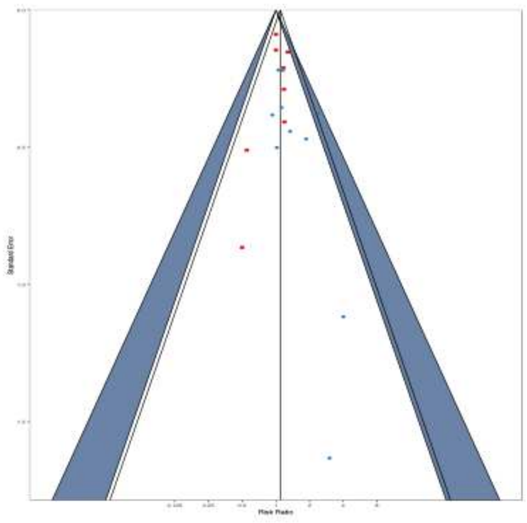

D. Efficacy Endpoint:
Stent thrombosis

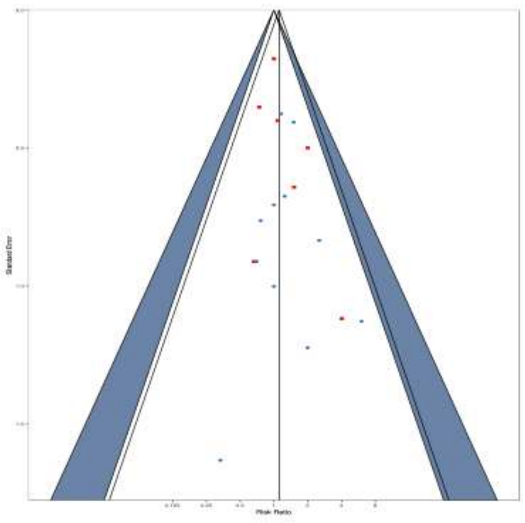

E. Efficacy Endpoint:
Coronary revascularisation

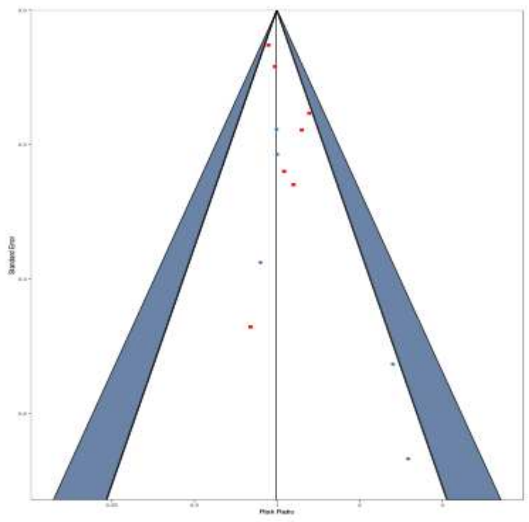

F. Efficacy Endpoint:
Stroke

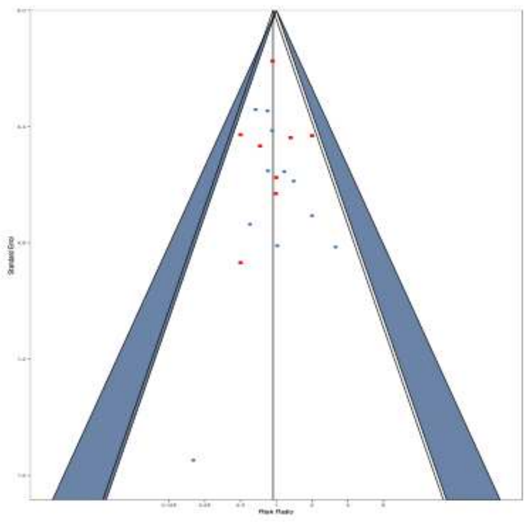

Bularga A, *et al. Open Heart* 2021; 8:e001707. doi: 10.1136/openhrt-2021-001707

G. Safety Endpoint:
Any bleeding

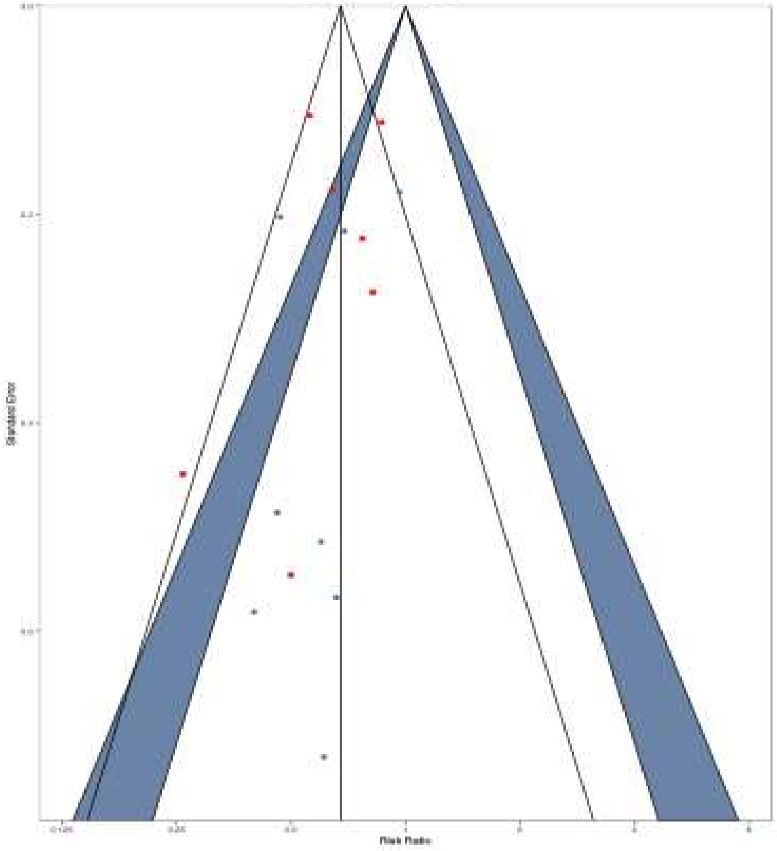

H. Safety Endpoint:
Major bleeding

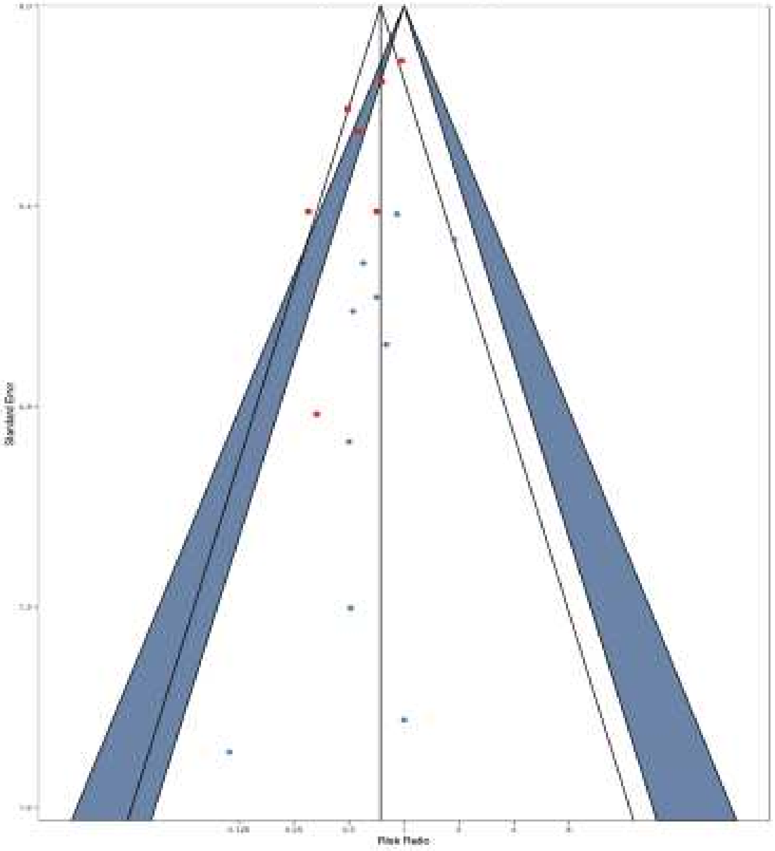

5

S3 Figure. Pooled risk estimates for each outcome of interest

*(*Studies included in sensitivity analysis [≤3 months vs 12 months])*

A. *All-cause mortality (19 trials)*

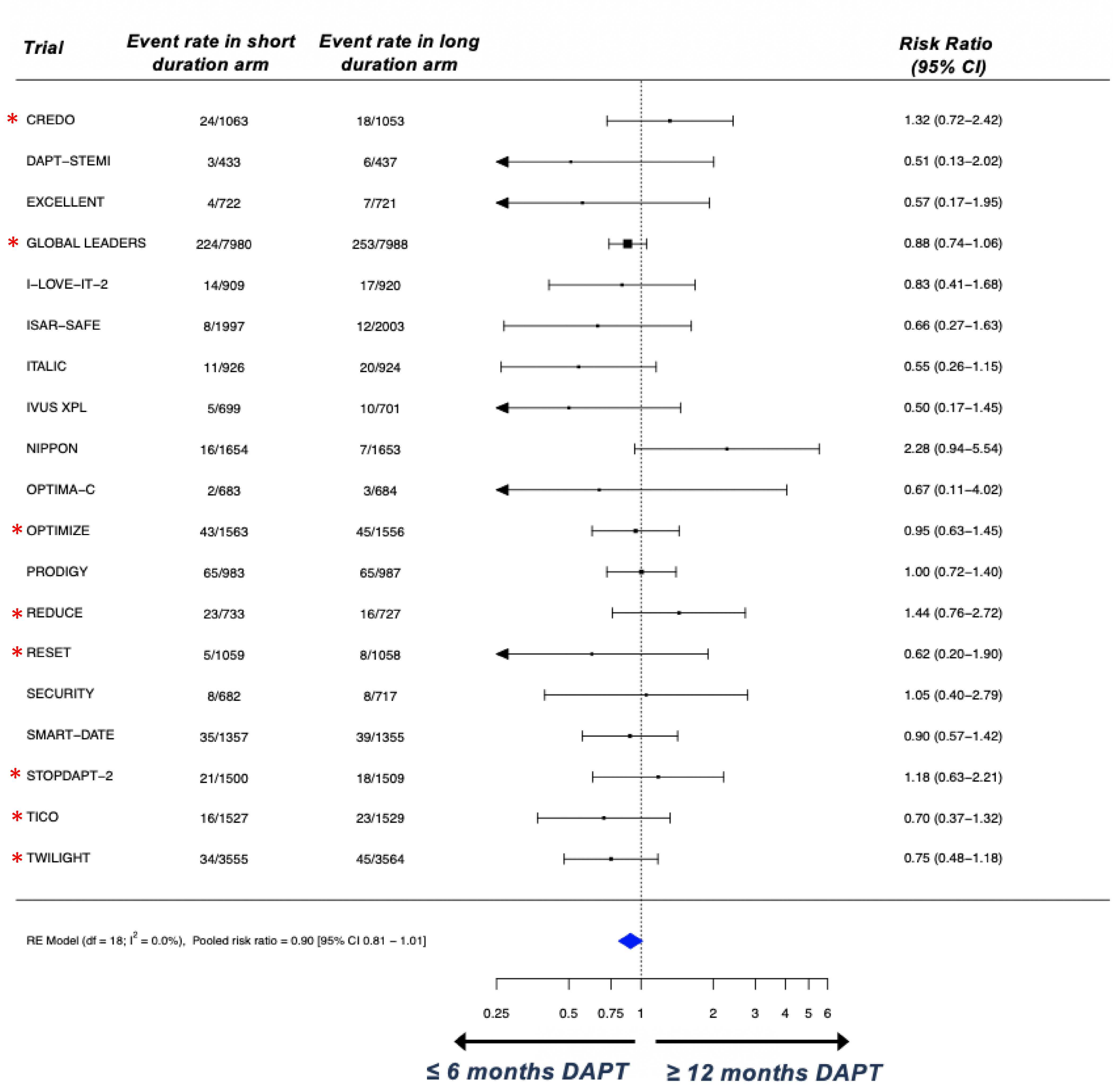

B. Efficacy Endpoint: *Cardiac death (16 trials)*

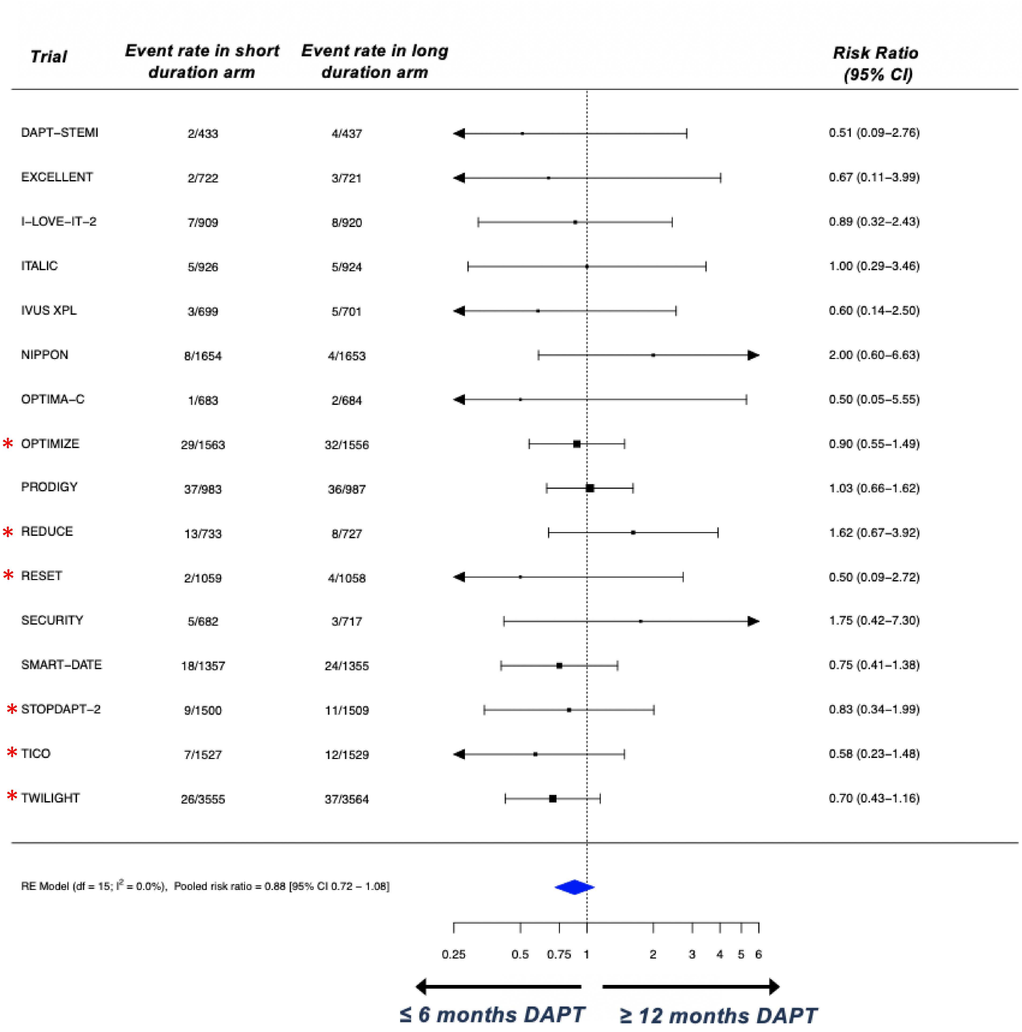

C. Efficacy Endpoint: *Myocardial Infarction (18 trials)*

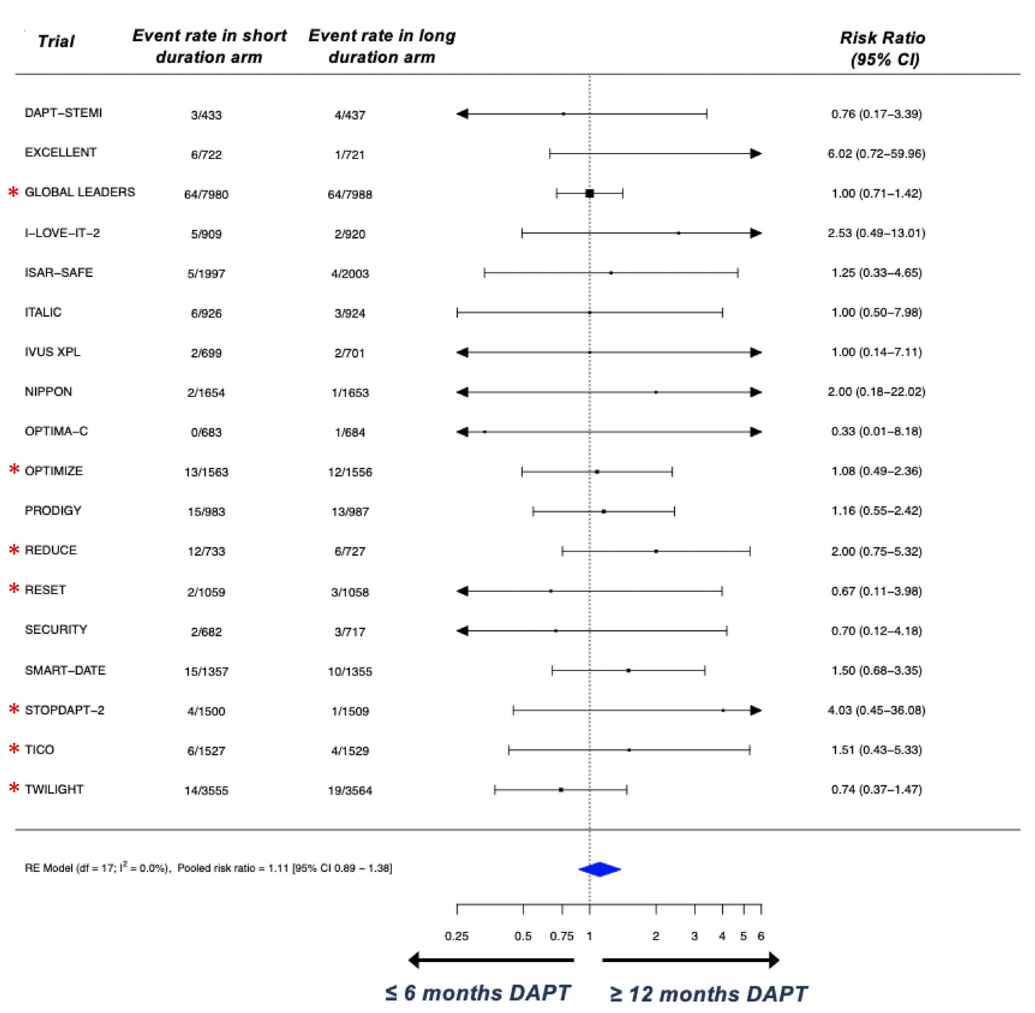

D. Efficacy Endpoint: *Stent thrombosis (18 trials)*

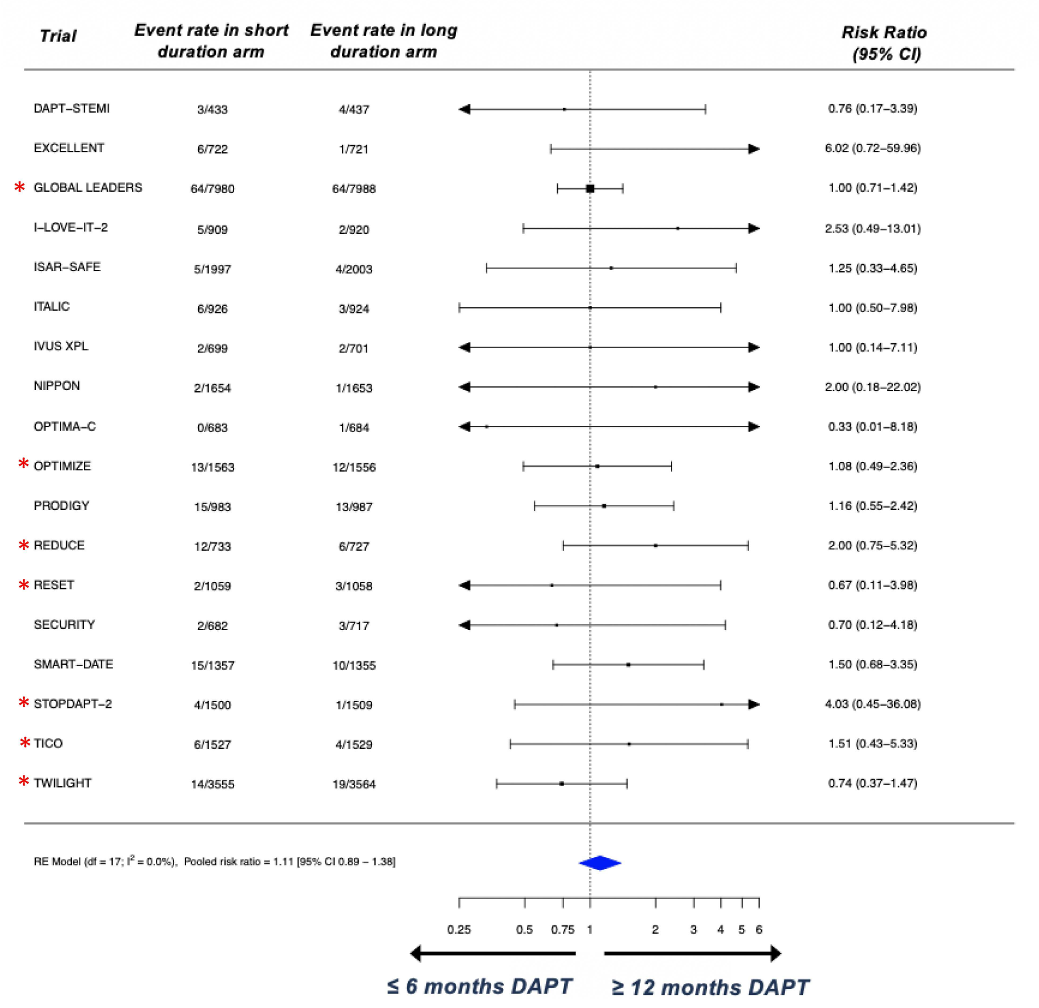

E. Efficacy Endpoint: *Coronary revascularisation (12 trials)*

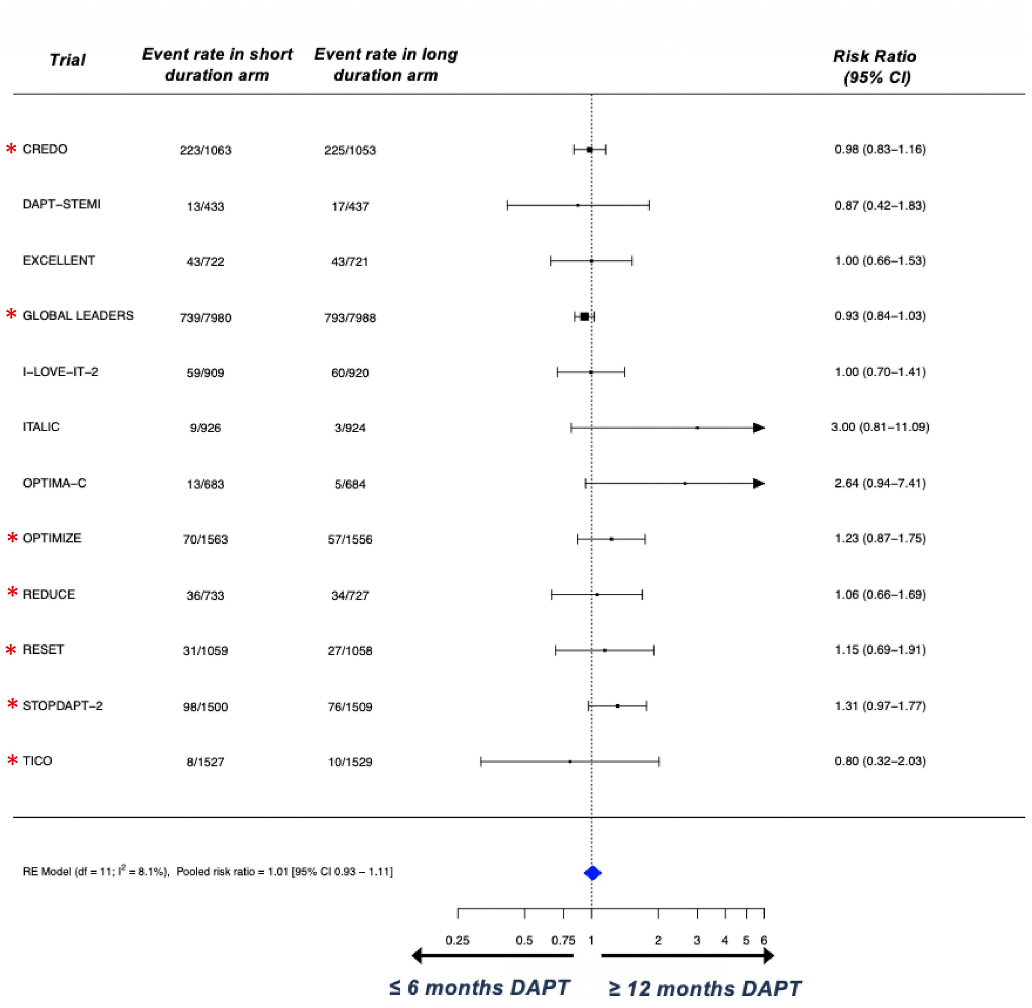

F. Efficacy Endpoint: *Stroke (19 trials)*

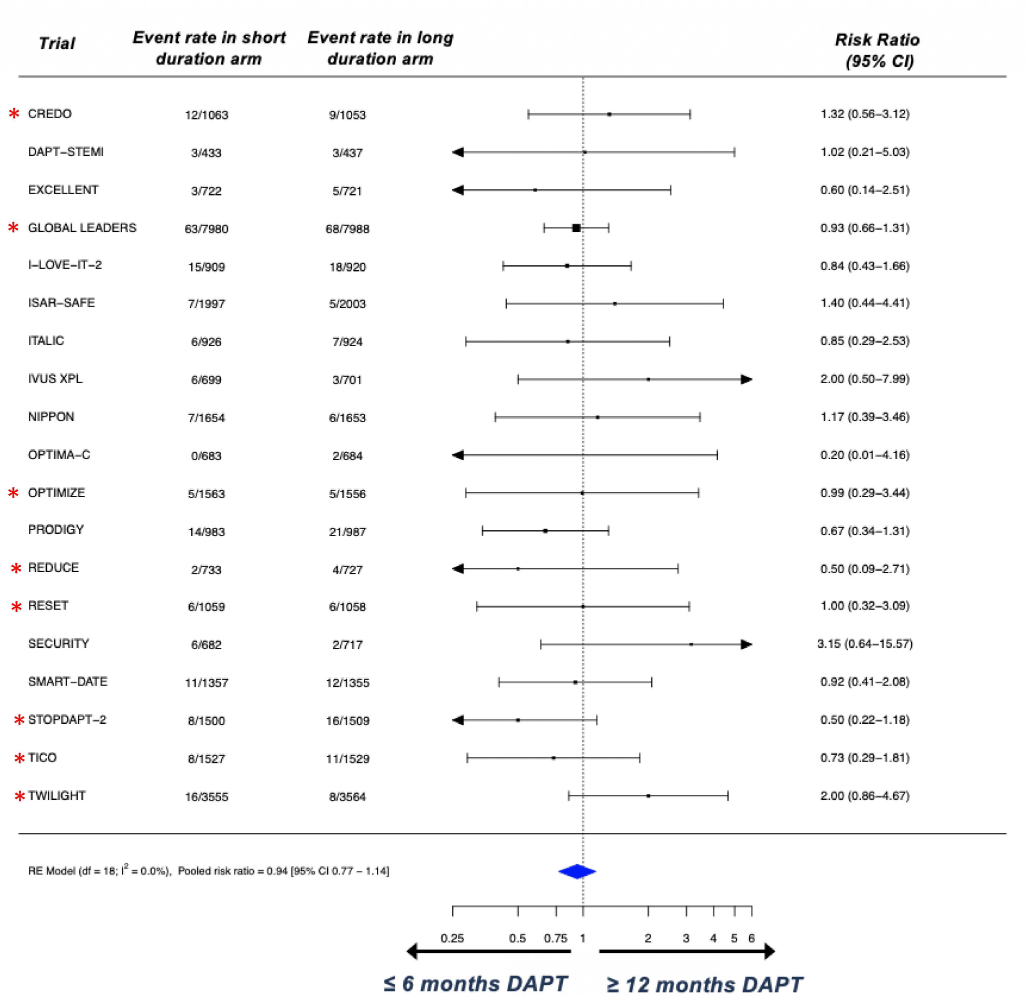

G. Safety Endpoint: *Any bleeding (15 trials)*

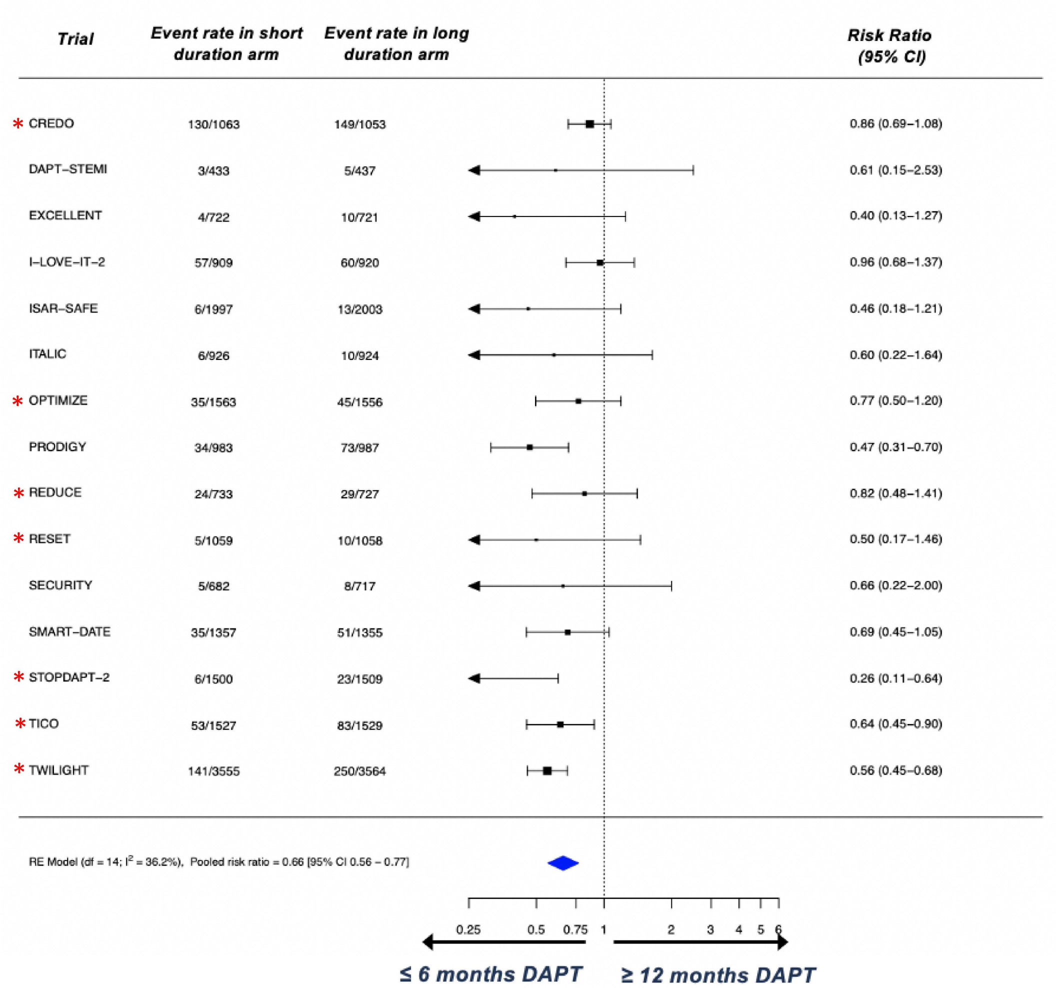

H. Safety Endpoint: *Major bleeding (18 trials)*

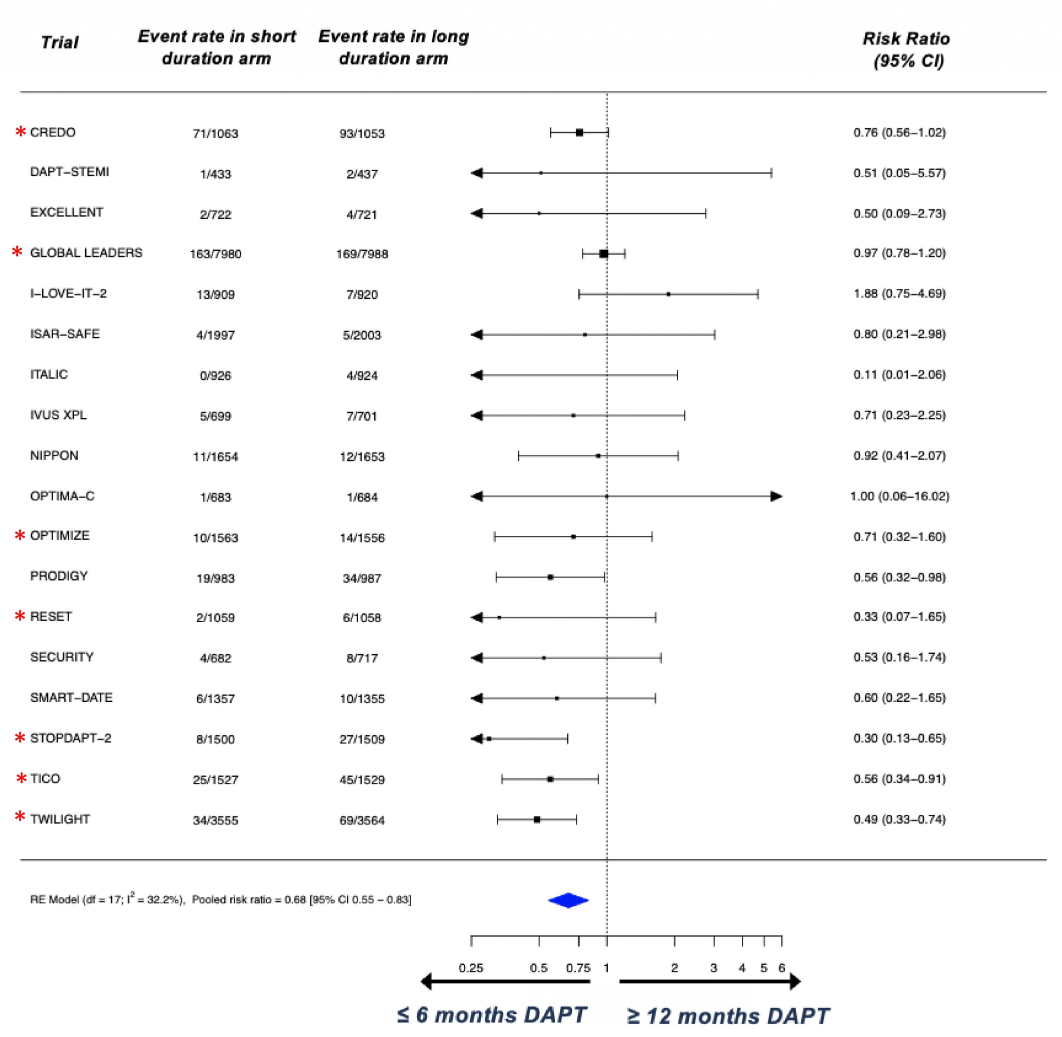

S4 Figure. Panel of two forest plots showing pooled risk estimates according to outcomes of interest in a sensitivity analysis. Panel A shows analysis restricted to studies (n=11) in which the P2Y12 receptor antagonist was clopidogrel only. Panel B shows analysis restricted to trials (n=8) in which the P2Y12 receptor antagonist was clopidogrel or prasugrel or ticagrelor.

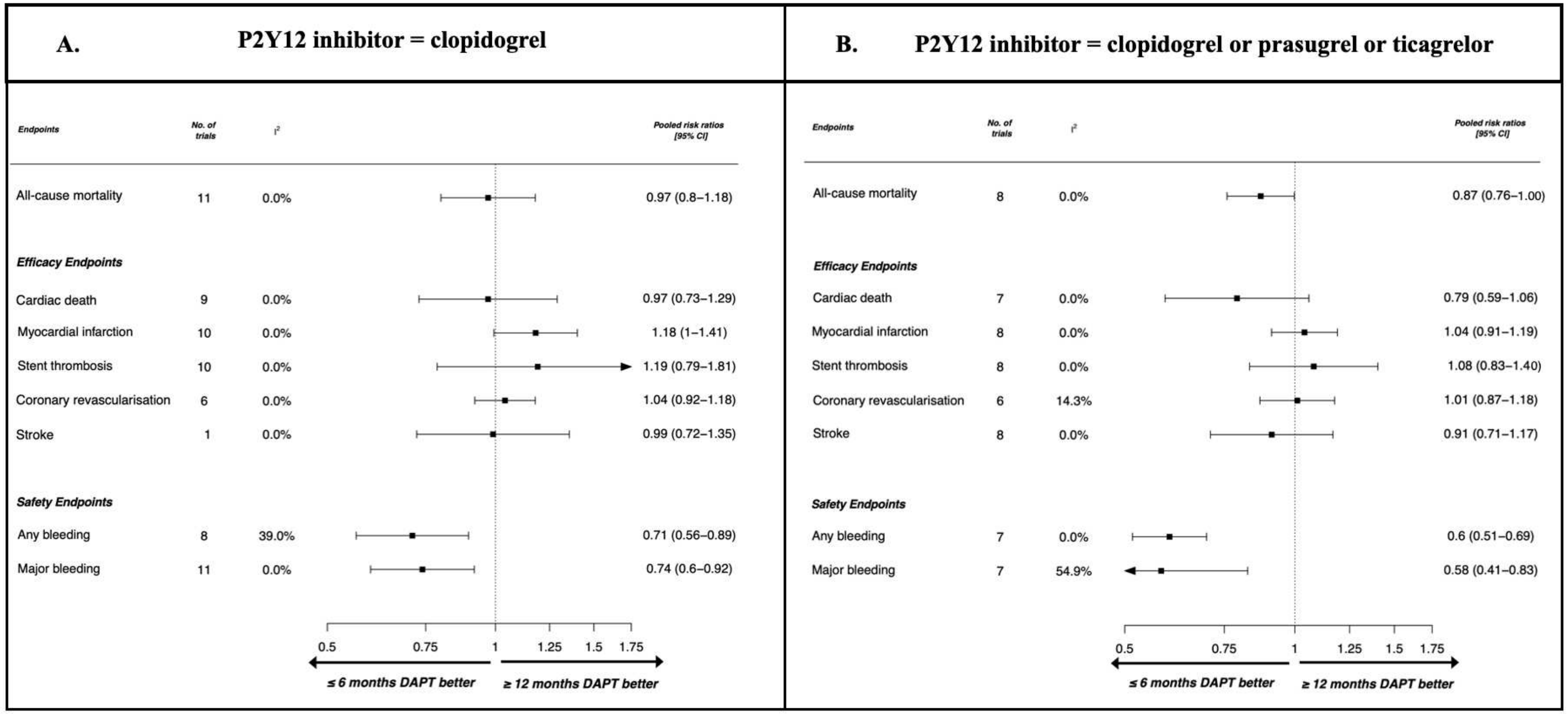

S1 Table: Search criteria

Medline

ID	SEARCH
#1	drug eluting stent/
#2	(eluting adj stent$).ab,ti.
#3	(coronary adj3 angioplast$).ab,ti.
#4	(coronary adj2 dilatation$).ab,ti.
#5	coronary stent$.ab,ti.
#6	Percutaneous Coronary Intervention/
#7	(percutaneous coronary adj2 (interven$ or revascular$)).ab,ti.
#8	PCI.ab,ti.
#9	coronary atherectom$.ab,ti.
#10	exp Coronary Artery Disease/
#11	exp coronary artery/
#12	exp angina pectoris/
#13	coronary.tw.
#14	angina.tw.
#15	myocardial infarction.tw.
#16	thienopyridine derivative/
#17	Platelet Aggregation Inhibitors/
#18	(antiplatelet$ or anti-platelet$ or antithrombocytic or anti-thrombocytic).ab,ti.
#19	(cyclooxygenase inhibitor$ or thienopyridine$).ab,ti.
#20	(thromboxane A2 adj3 (inhib$ or antag$)).ab,ti.
#21	aspirin/
#22	(clopidogrel or ticagrelor or prasugrel).ab,ti.
#23	dual antiplatelet.ab,ti.
#24	letter/
#25	editorial/
#26	news/
#27	exp historical article/
#28	Anecdotes as topic/
#29	comment/
#30	case report/
#31	(letter or comment$).ti.
#32	randomized controlled trial/ or Randomized Controlled Trials as Topic/ or random$.ti,ab.
#33	animals/ not humans/
#34	exp Animals, Laboratory/
#35	exp Animal Experimentation/
#36	exp Models, Animal/
#37	exp rodentia/
#38	(rat or rats or mouse or mice).ti.
#39	meta analysis as topic/
#40	meta analysis/
#41	(meta analy$ or metaanaly$ or metanaly$ or meta regression).ti,ab.

#42	((systematic$ or evidence$) adj2 (review$ or overview$)).ti,ab.
#43	(reference list$ or bibliograph$ or hand search$ or manual search$ or relevant journals).ab.
#44	(search strategy or search criteria or systematic search or study selection or data extraction).ab.
#45	(search$ adj4 literature).ab.
#46	(medline or pubmed or cochrane or embase or psychlit or psyclit or psychinfo or cinahl or science citation index or bids or cancerlit).ab.
#47	cochrane.jw.
#48	((multiple treatment$ or indirect or mixed) adj2 comparison).ti,ab.
#49	randomized controlled trial.pt. or randomized controlled trial/ or Randomized Controlled Trials as Topic/
#50	controlled clinical trial.pt.
#51	randomi#ed.ab.
#52	placebo.ab.
#53	randomly.ab.
#54	clinical trials as topic.sh.
#55	trial.ti.
#56	1 or 2 or 3 or 4 or 5 or 6 or 7 or 8 or 9 or 10 or 11 or 12 or 13 or 14 or 15
#57	16 or 17 or 18 or 19 or 20 or 21 or 22 or 23
#58	56 and 57
#59	24 or 25 or 26 or 27 or 28 or 29 or 30 or 31
#60	59 not 32
#61	33 or 34 or 35 or 36 or 37 or 38 or 60
#62	58 not 61
#63	39 or 40 or 41 or 42 or 43 or 44 or 45 or 46 or 47 or 48
#64	49 or 50 or 51 or 52 or 53 or 54 or 55
#65	63 or 64
#66	62 and 65

Central

ID	Search
#1	MeSH descriptor: [Drug-Eluting Stents] this term only
#2	(eluting near stent*):ti,ab,kw
#3	(coronary near/3 angioplast*):ti,ab,kw
#4	(coronary near/2 dilatation*):ti,ab,kw
#5	coronary stent*:ti,ab,kw
#6	[mh "Percutaneous Coronary Intervention"]
#7	(percutaneous coronary near/2 (interven* or revascular*)):ti,ab,kw
#8	PCI:ti,ab,kw
#9	coronary artery atherectom*:ti,ab,kw
#10	MeSH descriptor: [Coronary Artery Disease] explode all trees
#11	coronary artery obstruction
#12	MeSH descriptor: [Coronary Vessels] explode all trees
#13	heart infarction
#14	MeSH descriptor: [Angina Pectoris] explode all trees
#15	coronary
#16	angina
#17	myocardial infarction
#18	{or #1-#17}
#19	[mh "Platelet aggregation inhibitors"]
#20	[mh "Cyclooxygenase Inhibitors"] or [mh Thienopyridines] or [mh "Thromboxane A2"/AI] or 3 [mh "Purinergic P2Y Receptor Antagonists"]
#21	antiplatelet* or anti-platelet* or antithrombocytic or "anti-thrombocytic":ti,ab,kw
#22	"thromboxane A2" near/3 (inhib* or antag*):ti,ab,kw
#23	aspirin*:ti,ab,kw
#24	clopidogrel or ticagrelor or prasugrel:ti,ab,kw
#25	dual antiplatelet:ti,ab,kw
#26	{or #19-#25}
#27	#18 and #26
#28	[mh animals] not [mh humans]
#29	rat or rats or mouse or mice:ti,ab,kw
#30	#28 or #29
#31	#27 not #30
#32	randomized:ti,ab,kw
#33	placebo:ti,ab,kw
#34	randomly:ti,ab,kw
#35	clinical trials:ti,ab,kw
#36	{or #32-#35}
#37	#31 and #36

Web of Science

SET	SEARCH
#1	(TS=drug eluting stent)
#2	(TS=(eluting near stent*))
#3	(TS=(coronary near/3 angioplast*))
#4	(TS=(coronary near/2 dilatation*))
#5	(TS=coronary stent*)
#6	(TS="Percutaneous Coronary Intervention")
#7	(TS=(percutaneous coronary near/2 (interven* or revascular*)))
#8	(TS=PCI)
#9	(TS=coronary atherectom*)
#10	(TS="Platelet aggregation inhibitors")
#11	(TS="thienopyridine derivative")
#12	(TS=(antiplatelet* or anti-platelet* or antithrombocytic or anti-thrombocytic))
#13	(TS=(cyclooxygenase inhibitor* or thienopyridine*))
#14	(TS=(thromboxane A2 near/3 (inhib* or antag*)))
#15	(TS=aspirin)
#16	(TS=(clopidogrel or ticagrelor or prasugrel))
#17	(TS="dual antiplatelet*")
#18	#17 OR #16 OR #15 OR #14 OR #13 OR #12 OR #11 OR #10
#19	(TS= clinical trial* OR TS=research design OR TS=comparative stud* OR TS=evaluation stud* OR TS=controlled trial* OR TS=follow-up stud* OR TS=prospective stud* OR TS=random* OR TS=placebo* OR TS=(single blind*) OR TS=(double blind*))
#20	TS=cardia* infarct*
#21	TS=myocardial infarct*
#22	TS=heart infarct*
#23	TS=heart attack*
#24	TS=(coronary SAME syndrome*)
#25	#24 OR #23 OR #22 OR #21 OR #20 OR #9 OR #8 OR #7 OR #6 OR #5 OR #4 OR #3 OR #2 OR #1
#26	#25 AND #19 AND #18

Embase

ID	SEARCH
#1	exp stent/
#2	stent*.tw.
#3	exp drug eluting stent/
#4	drug elut*.tw.
#5	eluting stent*.tw.
#6	exp percutaneous coronary intervention/
#7	balloon angioplast*.tw.
#8	(percutaneous adj6 coronary intervention*).tw.
#9	PCI.tw.
#10	(intervention* adj6 percutaneous coronary).tw.
#11	(revascularization* adj6 percutaneous coronary).tw.
#12	(angioplast* adj6 coronary).tw.
#13	percutaneous coronary.tw.
#14	((transluminal or trans-luminal) adj6 coronary).tw.
#15	exp heart muscle ischemia/
#16	((myocardial or myocardium or subendocardial or transmural or cardiac or cardial or coronary or heart) adj2 (infarct* or postinfarct* or hypoxi* or anoxi* or failure* or decompensation or insufficien*)).tw.
#17	(heart disease* or coronary disease* or IHD or CIHD or CHD).tw.
#18	(myocardial dysfunction or angina or stenocardia).tw.
#19	((ischemi* or ischaemi*) adj2 (myocardium or myocardial or heart or coronary or cardiac or cardial or subendocardial or cardiomyopath*)).tw.
#20	((artery occlusion* or artery disease* or arterioscleros* or atheroscleros*) adj2 coronary).tw.
#21	exp acute coronary syndrome/
#22	exp heart infarction/
#23	coronary artery thrombosis/
#24	coronary thrombosis.tw.
#25	acute coronary.tw.
#26	exp unstable angina pectoris/
#27	myocardial infarct*.tw.
#28	heart infarct*.tw.
#29	acs.tw.
#30	ami.tw.
#31	(coronary adj3 syndrome*).tw.
#32	acute angina.tw.
#33	(unstable adj3 angina).tw.
#34	unstable coronary.tw.
#35	(anti?platelet* or anti platelet* or anti?thrombo* or anti thrombo* or aspirin* or clopidogrel or prasugrel or ticagrelor or ticlopidine or (platelet and aggregation and inhibitor*) or (anti and thrombosis)).mp.
#36	antithrombocytic agent/ or acetylsalicylic acid/
#37	thienopyridine derivative/
#38	thienopyridine$.tw.
#39	(clopidogrel or prasugrel or ticagrelor).tw.

Bularga A, *et al. Open Heart* 2021; 8:e001707. doi: 10.1136/openhrt-2021-001707

#40	(ADP adj3 receptor antagonist*).tw,ot.
#41	1 or 2 or 3 or 4 or 5 or 6 or 7 or 8 or 9 or 10 or 11 or 12 or 13 or 14 or 15 or 16 or 17 or 18 or 19 or 20 or 21 or 22 or 23 or 24 or 25 or 26 or 27 or 28 or 29 or 30 or 31 or 32 or 33 or 34
#42	Randomized Controlled Trial/
#43	double-blind method/
#44	single-blind method/
#45	randomized controlled trial:.mp.
#46	controlled clinical trial/
#47	Clinical Trial/
#48	exp comparative study/
#49	follow up/
#50	prospective study/
#51	random:.mp.
#52	(random: adj5 (controlled or clinical)).mp.
#53	47 or 48 or 49 or 50
#54	51 or 52
#55	53 and 54
#56	42 or 43 or 44 or 45 or 46 or 55
#57	35 or 36 or 37 or 38 or 39 or 40
#58	41 and 56 and 57

S2 Table. Baseline characteristics for each study cohort stratified by DAPT duration

Study	*DAPT duration*	*Total population*	*Age, years (mean)*	*Male (%)*	*Diabetes*	*Hypertension*	*Hypercholesterolaemia*	*Current smoker*	*Previous MI*	*Previous stroke*	*Chronic kidney disease*
CREDO *(multi centre)*	1	1063	61.8	766 (72%)	270	740	780	313	353	74	NR
	12	1053	61.5	744 (71%)	290	710	800	339	366	67	NR
DAPT-STEMI *(multi centre)*	6	433	59.8	337 (78%)	54	193	120	218	26	14	NR
	12	437	60.2	332 (76%)	61	195	125	205	20	8	NR
EXCELLENT *(multi centre)*	6	722	63.0	470 (65%)	272	525	543	198	47	47	6
	12	721	62.4	461 (64%)	278	532	550	186	27	48	9
GLOBAL LEADERS *(multi centre)*	1	7980	64.5	6115 (77%)	2049	5882	5345	2066	1831	210	1099
	12	7988	64.6	6139 (77%)	1989	5833	5423	2103	1879	211	1072
I-LOVE-IT-2 *(multi centre)*	6	909	60.4	611 (67%)	211	554	230	333	156	84	NR
	12	920	60.0	632 (69%)	203	596	215	352	145	87	NR
ISAR-SAFE *(multi centre)*	6	1997	67.2	1611 (80%)	495	1797	1747	995	516	NR	NR
	12	2003	67.2	1612 (80%)	484	1830	1748	1025	491	NR	NR
ITALIC *(multi centre)*	6	926	61.6	750 (80%)	336	603	625	473	144	28	29
	24	924	61.5	733 (79%)	349	594	618	487	138	26	25
IVUS-XPL *(multi centre)*	6	699	63	470 (67%)	22	443	473	171	34	NR	NR
	12	701	64	494 (70%)	23	455	456	165	29	NR	NR
NIPPON *(multi centre)*	6	1654	67.4	1304 (79%)	619	1177	1130	960	201	48	NR
	18	1653	67.2	1312 (79%)	635	1209	1132	997	195	41	NR
OPTIMA-C *(multi centre)*	6	684	62.8	478 (70%)	199	426	204	184	18	NR	NR
	12	683	64.4	464 (68%)	203	437	195	184	25	NR	NR
OPTIMIZE *(multi centre)*	3	1563	61.3	992 (63%)	554	1350	953	290	541	38	114
	12	1556	61.9	982 (63%)	549	1371	952	269	542	38	89
PRODIGY *(multi centre)*	6	983	67.9	747 (76%)	233	693	525	247	258	39	NR
	24	987	67.8	764 (77%)	244	721	533	222	270	37	NR

Bularga A, *et al. Open Heart* 2021; 8:e001707. doi: 10.1136/openhrt-2021-001707

REDUCE *(multi centre)*	3	733	61	620 (85%)	162	379	346	313	94	11	NR
	12	727	60	576 (79%)	145	375	333	314	88	15	NR
RESET *(multi centre)*	3	1059	62.4	682 (64%)	316	660	611	267	19	NR	NR
	12	1058	62.4	665 (63%)	305	650	634	241	17	NR	NR
SECURITY *(multi centre)*	6	682	64.9	529 (78%)	206	508	446	378	145	NR	NR
	12	717	65.5	551 (77%)	223	510	436	410	144	NR	NR
SMART-DATE *(multi centre)*	6	1357	62.0	1016 (75%)	365	669	322	506	30	52	13
	12	1355	62.2	1028 (76%)	379	654	336	536	23	58	6
STOPDAPT-2 *(multi centre)*	1	1500	68.1	1183 (79%)	585	1105	1116	399	207	NR	82
	12	1509	69.1	1154 (76%)	574	1116	1128	311	199	NR	84
TICO *(multi centre)*	3	1527	61.0	1204 (79%)	418	760	NR	555	64	60	292
	12	1529	61.0	1224 (80%)	417	781	NR	587	49	66	328
TWILIGHT *(multi centre)*	3	3555	65.2	2709 (76%)	1319	2580	2157	726	1496	NR	572
	12	3564	65.1	2712 (76%)	1301	2574	2146	822	1020	NR	573
NR = not recorded											

S3 Table. Endpoint definitions

A. Efficacy Endpoints

Study	*Cardiac death*	*Myocardial infarction*	*Stent thrombosis*	*Coronary revascularisation*	*Stroke*
CREDO	Outcome not reported	Outcome not reported	Outcome not reported	Urgent target vessel revascularization was defined as CABG initiated within 24 hours of the index procedure due to an inadequate or unstable result of the index procedure; repeat PCI or CABG of the target vessel initiated within 1 week of (re)hospitalization for acute MI or unstable angina; or repeat PCI or CABG of the culprit vessel initiated within 24 hours of the last episode(s) of ischemia.	Stroke was defined as a new focal neurologic deficit of vascular origin lasting at least 24 hours.
DAPT-STEMI	Any death due to immediate cardiac cause (myocardial infarction, low-output failure, fatal arrhythmia). Unwitnessed death and death of unknown cause will be classified as cardiac death.	ARC criteria*	The presence of a thrombus that originates in the stent or in the segment 5mm proximal or distal to the stent and presence of at least 1 of the following criteria within a 48-hour time window: acute onset of ischemic symptoms at rest; new ischemic ECG changes that suggest acute ischemia; typical rise and fall in cardiac biomarkers.	Any clinically indicated revascularization procedure (PCI or CABG) not foreseen at randomization will be considered as a revascularization event.	Acute neurological event of at least 24 hours of duration, with focal signs and symptoms and without evidence supporting any alternative explanation. Diagnosis of stroke requires confirmation by computed tomography (CT) or magnetic resonance imaging (MRI) or pathological confirmation
EXCELLENT	All deaths were considered cardiac unless a definite noncardiac cause could be established.	ARC criteria*	ARC criteria*	Target lesion revascularization was defined as repeat revascularization of the treated vessel by PCI or CABG.	Stroke, as detected by the occurrence of a new neurological deficit, was confirmed by a neurologist and imaging.
GLOBAL LEADERS	Outcome not reported	3rd Universal Definition of Myocardial Infarction	ARC criteria*	ARC criteria*	Rapid onset of focal/global neurological deficit. Confirmation by at least one of: a neurologist/neurosurgeon or neuroimaging (CT, MRI or Angio) or lumbar puncture.
I-LOVE-IT-2	ARC criteria*	ARC criteria*	ARC criteria*	Revascularization for a stent stenosis.	ARC criteria*
ISAR-SAFE	Outcome not reported	TIMI criteria	ARC criteria*	Outcome not reported	Confirmation by CT, MRI or pathology.
ITALIC	Any death due to proximate cardiac cause (MI, low-output failure, fatal arrhythmia), unwitnessed death and death of unknown cause, and all procedure-related deaths, will be classified as cardiac death.	ARC criteria*	ARC criteria*	TVR is defined as any repeat percutaneous intervention or surgical bypass of any segment of the target vessel.	ARC criteria*
IVUS-XPL	All deaths were considered cardiac deaths unless a definite noncardiac cause could be established.	Presence of consistent clinical symptoms, electrocardiographic changes, or abnormal imaging findings, combined with a creatine kinase myocardial band fraction increase greater than the upper normal limit or an increase in troponin T or troponin I to >99th percentile of the upper normal limit.	ARC criteria*	Outcome not reported	Occurrence of a new neurological deficit, was confirmed using a neurological examination and imaging studies.
NIPPON	Any death due to an immediate cardiac cause (myocardial infarction, low-output failure, or fatal arrhythmia). Unwitnessed death and	Classified as Q-wave (new pathological Q waves in 2 or more continuous ECG leads) or non-Q-wave and when myocardial	ARC criteria*	Outcome not reported	Defined as the sudden onset of vertigo, numbness, dysphasia, weakness, visual field defects, dysarthria or other focal

	death of unknown cause was classified as cardiac death. This included all procedure-related deaths, including those related to concomitant treatment.	enzymes are at or above the upper limit of normal, it should be considered as MI at registration, and when the serum level of troponin or CK-MB exceeds the upper limit of normal more than 48 hours after PCI or within 72 hours after CABG.			neurological deficits due to vascular lesions of the brain such as haemorrhage, embolism, thrombosis, or ruptured aneurysm.
OPTIMA-C	ARC criteria*	ARC criteria*	Definition unavaialble	ARC criteria*	ACR criteria*
OPTIMIZE	ARC criteria*	WHO criteria	ARC criteria*	Target vessel revascularization was a new percutaneous or surgical reintervention (CABG) of a target vessel	Acute neurological event with duration of 24 hours with confirmation by either computed tomography or magnetic resonance imaging or pathological study.
PRODIGY	ARC criteria*	ARC criteria*	ARC criteria*	Outcome not reported	ARC criteria*
REDUCE	ARC criteria*	3rd Universal Definition of Myocardial Infarction	ARC criteria*	Definition unavailable	Definition unavailable
RESET	ARC criteria *	ARC criteria*	ARC criteria*	ARC criteria*	ARC criteria*
SECURITY	Any death without a non-cardiac cause.	Cardiac enzyme elevation above the upper normal limit associated with at least one ischaemic symptom; development of Q waves on the electrocardiogram; electrocardiogram changes indicative of ischaemia or coronary artery intervention.	ARC criteria*	Outcome not reported	any new neurological deficit lasting >24 hours associated with neuroimaging evidence (CT or MRI)
SMART-DATE	All deaths were considered cardiac unless a definite non-cardiac cause could be established.	Elevated cardiac enzymes (cardiac troponin or myocardial band fraction of creatine kinase) above the upper reference limit with ischaemic symptoms or electrocardiography findings indicative of ischaemia that was not related to the index procedure.	ARC criteria*	Outcome not reported	Stroke was defined as any non-convulsive focal or global neurological deficit of abrupt onset lasting more than 24h or leading to death, which was caused by ischaemia or haemorrhage.
STOPDAPT-2	ARC criteria*	ARC criteria*	ARC criteria*	ARC criteria*	Acute onset of a neurological deficit that persists for at least 24 hours and is the result of a disturbance of the cerebral circulation due to ischemia or haemorrhage. Deficits that last ² 24 hours are due to transient ischemic neurological attack and are not classified in this category.
TICO	ARC criteria*	Definition unavailable	ARC criteria*	ARC criteria*	Definition unavailable
TWILIGHT	Any death due to proximate cardiac cause (MI, low- output failure, fatal arrhythmia), unwitnessed death and death of unknown cause, all procedure-related deaths including those related to concomitant treatment, will be classified as cardiac death.	3rd Universal Definition of Myocardial Infarction	ARC criteria*	Outcome not reported	Stroke is defined as an acute symptomatic episode of neurological dysfunction, more than 24 hours in duration in the absence of therapeutic intervention or death, due to cerebral, spinal or retinal tissue injury as evidenced by neuroimaging or lumbar puncture.

Bularga A, *et al. Open Heart* 2021; 8:e001707. doi: 10.1136/openhrt-2021-001707

B. Safety Endpoints

Study	*Any bleeding*	*Major bleeding*
CREDO	Outcome not reported	TIMI major
DAPT-STEMI	TIMI criteria	TIMI major
EXCELLENT	TIMI criteria	TIMI major
GLOBAL LEADERS	BARC criteria	BARC 3 or 5
I-LOVE-IT-2	BARC criteria	BARC 3, 4 or 5
ISAR-SAFE	TIMI criteria	TIMI major
ITALIC	TIMI criteria	TIMI major
IVUS-XPL	Outcome not reported	TIMI major
NIPPON	Outcome not reported	BARC 3 or 5
OPTIMA-C	Outcome not reported	TIMI major
OPTIMIZE	Major bleeding plus bleeding events that did not meet criteria for either major or severe or life-threatening bleeding according to modified major REPLACE-2 and severe or life-threatening GUSTO criteria.	Modified major REPLACE -2 and severe or life-threatening GUSTO.
PRODIGY	BARC criteria	BARC 3 or 5
REDUCE	BARC 2, 3 or 5	Outcome not reported
RESET	TIMI criteria	TIMI major
SECURITY	BARC criteria	BARC 3 or 5
SMART-DATE	BARC criteria	BARC 3, 4 or 5
STOPDAPT-2	BARC criteria	BARC 3 or 5
TICO	TIMI criteria	TIMI major
TWILIGHT	BARC criteria	BARC 3 or 5
*ARC = Academic Research Consortium; BARC = Bleeding Academic Research Consortium; GUSTO = Global Use of Strategies to Open Occluded Arteries; REPLACE = Randomized Evaluation of PCI Linking Angiomax to Reduced Clinical Events; TIMI = Thrombolysis in Myocardial Infarction.		

S4 Table. Risk of bias assessment

Study	*Random sequence generation*	*Allocation concealment*	*Blinding of participants and personnel*	*Blinding of outcome assessment*	*Incomplete outcome data*	*Selective reporting*	*Other bias*
CREDO	+	+	+	+	+	+	+
DAPT-STEMI	+	?	±	+	+	+	+
EXCELLENT	+	±	±	+	+	+	+
GLOBAL LEADERS	+	+	±	+	+	+	+
I-LOVE-IT-2	+	+	±	+	+	+	+
ISAR-SAFE	+	+	+	+	+	+	+
ITALIC	+	+	±	?	±	+	±
IVUS-XPL	+	+	±	+	+	±	+
NIPPON	+	+	±	+	+	+	+

Bularga A, *et al. Open Heart* 2021; 8:e001707. doi: 10.1136/openhrt-2021-001707

OPTIMA-C	+	+	±	+	+	+	+
OPTIMIZE	+	?	±	+	+	+	+
PRODIGY	+	+	±	+	+	+	+
REDUCE	+	+	±	+	+	±	+
RESET	+	+	±	±	+	+	+
SECURITY	+	+	±	±	+	+	+
SMART-DATE	+	+	±	+	+	+	+
STOPDAPT-2	+	+	±	+	+	+	+
TICO	+	?	±	?	+	+	+
TWILIGHT	+	+	+	+	+	+	+

+ low risk of bias; ± some risk of bias; - high risk of bias; ? unable to ascertain risk of bias

Bularga A, *et al. Open Heart* 2021; 8:e001707. doi: 10.1136/openhrt-2021-001707

Supplement Appendix

Duration of dual antiplatelet therapy

and stability of coronary heart disease:

a 60,000-patient meta-analysis of randomised controlled trials

STATISTICAL ANALYSIS PLAN

Version 1. January 2020
Version 2. June 2020

Bularga A, *et al. Open Heart* 2021; 8:e001707. doi: 10.1136/openhrt-2021-001707

Contents

Contents **29**

1 Introduction **30**

1.1 Background *30*

1.2 Study questions *5*

1.2.1 Study Objectives 5

2 Statistical analysis plan **5**

2.1 Objectives *5*

2.2 Study design *5*

2.2.1 Inclusion criteria 5

2.2.2 Selection of studies 6

2.2.3 Data extraction 6

3 List of analyses **7**

3.1 Data synthesis *7*

3.2 Risk of bias *7*

3.2.1 Publication bias 7

3.3 Subgroup analysis *7*

4 References **8**

1 Introduction

1.1 Background

Despite substantial evidence supporting the use of dual antiplatelet therapy in patients with acute coronary syndrome, there remains major uncertainty regarding the optimal duration of therapy. The current ESC guidelines on the management of an acute coronary syndrome recommend that 12 months of dual antiplatelet therapy with aspirin and a $P2Y_{12}$ inhibitor should be standard therapy irrespective of the acute coronary syndrome revascularisation strategy (ie. medical therapy, percutaneous coronary intervention or coronary artery bypass graft surgery).[1,2] The guidelines offer consideration of shorter duration dual antiplatelet therapy for patients with high-risk of bleeding and longer for those who have tolerated the combination of medication without side effects and bleeding events.

Previous systematic reviews and meta-analyses concluded that shorter durations of DAPT may be superior to standard care in most patients, with apparent small reductions in all-cause mortality.[3,4] This suggests that the risk of major bleeding outweighs any benefit gained from the reduction in future atherothrombotic events. These meta-analyses reviewed trials which, for the most part, evaluated DAPT following percutaneous coronary intervention with drug-eluting stents in patients with chronic coronary syndromes. Recently, there have been several large-scale randomised controlled trials evaluating shorter durations of DAPT (≤3 months) in the setting of acute coronary syndromes (ACS).[5-7]

We aim to perform an updated systematic review and meta-analysis comparing outcomes in long-term DAPT (≥12 months) with short-term (≤6 months) and shorter (≤3 months) durations of DAPT incorporating the latest randomised controlled trial evidence.

Bularga A, *et al. Open Heart* 2021; 8:e001707. doi: 10.1136/openhrt-2021-001707

1.2 Study questions

1.2.1 Study Objectives

To evaluate the safety and efficacy of short term (≤6-months) dual antiplatelet therapy (DAPT) versus long term (≥12-months DAPT) in patients undergoing percutaneous coronary intervention stratified by stable coronary heart disease and acute coronary syndrome.

2 Statistical analysis plan

2.1 Objectives

The objective of this SAP is to describe the statistical analyses contributing to the final report and primary publication(s) of the analyses described below.

2.2 Study design

This is a systematic review and meta-analysis of randomised control trials evaluating the duration of dual antiplatelet therapy in patients with chronic coronary syndrome following percutaneous coronary intervention and in patients with acute coronary syndrome irrespective of the management strategy following the index presentation.

2.2.1 Inclusion criteria

- Randomised controlled trials comparing different durations of dual antiplatelet therapy, irrespective of presentation (acute or chronic coronary syndromes), or the management strategy (percutaneous coronary intervention, coronary artery bypass graft surgery or medical therapy alone) that assessed at least one of the pre-specified outcomes of interest were included in this systematic review and meta-analysis.

Primary Outcome

- All-cause mortality

Secondary Efficacy Outcomes

- Cardiac death
- Myocardial infarction
- Stent thrombosis
- Coronary revascularisation
- Stroke

Secondary Safety Outcomes

- Any bleeding
- Major bleeding

- All randomised control trials evaluating duration of DAPT in patients with stable coronary disease or acute coronary syndrome and evaluating the above outcomes will be included in this analysis, irrespective of the management strategy following the acute coronary syndrome:

    - Percutaneous coronary intervention with drug-eluting stent (irrespective of type)
    - Percutaneous intervention with bare-metal stent
    - Medical therapy alone
    - Coronary artery bypass graft (CABG) surgery

2.2.2 Selection of studies

We will perform a systematic search of Embase, Medline, Central and Web of Science. All studies identified in the systematic literature search will be independently screened by two independent investigators and conflicts adjudicated by a third using a pre-specified protocol registered on PROSPERO. Where there are multiple articles from the same cohort, the article with the largest number of participants will be included.

2.2.3 Data extraction

Data extraction will be carried out independently by two investigators and conflicts will be adjudicated by a third.

3 List of analyses

3.1 Data synthesis

We will pool risk ratios and 95% confidence intervals to summarise each endpoint. We will assess between study heterogeneity and will calculate summary statistics using the random effects model for pairwise meta-analysis We will use forest plots to present the results of risk ratios and 95% confidence intervals. Adjusted funnel plot symmetry will be used to address potential publication bias. A prespecified sensitivity analysis evaluating studies which evaluated even shorter duration of DAPT (≤3 months) will be performed.

3.2 Risk of bias

3.2.1 Risk of bias in individual studies

We will assess each individual study for risk of bias across endpoint assessment and adjustment for confounders. The quality of the included studies will be assessed according to

the Cochrane Collaboration's tool[8] for assessing the risk of bias. Any discrepancies will be resolved by consensus, referring to the original articles and consulting with a third reviewer.

3.2 Subgroup analysis

Where possible, we will evaluate the relative risks in pre-specified subgroups according to index presentation: acute coronary syndrome and chronic coronary syndrome.

4 References for pre-specified analysis plan

1. Valgimigli M, Bueno H, Byrne RA, et al. 2017 ESC focused update on dual antiplatelet therapy in coronary artery disease developed in collaboration with EACTS. *Eur Heart J*. 2018;39:213-254.

2. Bhatt DL, Fox KA, Hacke W, *et al*; CHARISMA Investigators. Clopidogrel and aspirin versus aspirin alone for the prevention of atherothrombotic events. *N Engl J Med* 2006;354:1706–17.

3. Navarese EP, Andreotti F, Schulze V, Kolodziejczak M, Buffon A, Brouwer M, et al. Optimal duration of dual antiplatelet therapy after percutaneous coronary intervention with drug eluting stents: meta-analysis of randomised controlled trials. BMJ. 2015;350:h1618.

4. Yin SH, Xu P, Wang B, Lu Y, Wu QY, Zhou ML, et al. Duration of dual antiplatelet therapy after percutaneous coronary intervention with drug-eluting stent: systematic review and network meta-analysis. BMJ. 2019;365:l2222.

5. Vranckx P, Valgimigli M, Jüni P, Hamm C, Steg PG, Heg D, et al. Ticagrelor plus aspirin for 1 month, followed by ticagrelor monotherapy for 23 months vs aspirin plus clopidogrel or ticagrelor for 12 months, followed by aspirin monotherapy for 12 months after implantation of a drug-eluting stent: a multicentre, open-label, randomised superiority trial. The Lancet. 2018;392(10151):940-9.

6. Mehran R, Baber U, Sharma SK, Cohen DJ, Angiolillo DJ, Briguori C, et al. Ticagrelor with or without Aspirin in High-Risk Patients after PCI. N Engl J Med. 2019;381(21):2032-42.

7. Hahn J-Y, Song YB, Oh J-H, Cho D-K, Lee JB, Doh J-H, et al. 6-month versus 12-month or longer dual antiplatelet therapy after percutaneous coronary intervention in patients with acute coronary syndrome (SMART-DATE): a randomised, open-label, non-inferiority trial. The Lancet. 2018;391(10127):1274-84.

8. Savovic J, Weeks L, Sterne JA, Turner L, Altman DG, Moher D, et al. Evaluation of the Cochrane Collaboration's tool for assessing the risk of bias in randomized trials: focus groups, online survey, proposed recommendations and their implementation. Syst Rev. 2014;3:37.
